# Evaluation of clustering algorithms for protein-protein interaction networks

**DOI:** 10.1186/1471-2105-7-488

**Published:** 2006-11-06

**Authors:** Sylvain Brohée, Jacques van Helden

**Affiliations:** 1 Service de Conformation des Macromolécules Biologiques et de Bioinformatique. Université Libre de Bruxelles, CP 263, Campus Plaine, Bd. du Triomphe, B-1050 Bruxelles, Belgium

## Abstract

**Background:**

Protein interactions are crucial components of all cellular processes. Recently, high-throughput methods have been developed to obtain a global description of the interactome (the whole network of protein interactions for a given organism). In 2002, the yeast interactome was estimated to contain up to 80,000 potential interactions. This estimate is based on the integration of data sets obtained by various methods (mass spectrometry, two-hybrid methods, genetic studies). High-throughput methods are known, however, to yield a non-negligible rate of false positives, and to miss a fraction of existing interactions.

The interactome can be represented as a graph where nodes correspond with proteins and edges with pairwise interactions. In recent years clustering methods have been developed and applied in order to extract relevant modules from such graphs. These algorithms require the specification of parameters that may drastically affect the results. In this paper we present a comparative assessment of four algorithms: Markov Clustering (MCL), Restricted Neighborhood Search Clustering (RNSC), Super Paramagnetic Clustering (SPC), and Molecular Complex Detection (MCODE).

**Results:**

A test graph was built on the basis of 220 complexes annotated in the MIPS database. To evaluate the robustness to false positives and false negatives, we derived 41 altered graphs by randomly removing edges from or adding edges to the test graph in various proportions.

Each clustering algorithm was applied to these graphs with various parameter settings, and the clusters were compared with the annotated complexes.

We analyzed the sensitivity of the algorithms to the parameters and determined their optimal parameter values.

We also evaluated their robustness to alterations of the test graph.

We then applied the four algorithms to six graphs obtained from high-throughput experiments and compared the resulting clusters with the annotated complexes.

**Conclusion:**

This analysis shows that MCL is remarkably robust to graph alterations. In the tests of robustness, RNSC is more sensitive to edge deletion but less sensitive to the use of suboptimal parameter values. The other two algorithms are clearly weaker under most conditions.

The analysis of high-throughput data supports the superiority of MCL for the extraction of complexes from interaction networks.

## Background

Protein-protein interactions (PPI) play major roles in the cell: transient protein interactions are often involved in post-translational control of protein activity; enzymatic complexes ensure substrate channeling which drastically increases fluxes through metabolic pathways; large protein complexes play essential roles in basal cellular mechanisms such as DNA packaging (histones), transcription (RNA polymerase), replication (DNA polymerase), translation (ribosome), protein degradation (proteasome) ...

Various methods have been used to detect PPI. Co-immunoprecipitation, co-sedimentation, and two-hybrid systems have traditionally been used to characterize interactions at the level of a single protein complex. More recently, high-throughput methods have been developed for large-scale detection of pairwise interactions (two-hybrid systems, the split-ubiquitin method) [1-3] or multi-protein complexes (TAP-TAG, HMS-PCI) [4-7].

In 2002, von Mering *et al*. estimated that data resulting from combined experimental and computational approaches provide clues in favor of approximately 80,000 PPI in the yeast *Saccharomyces cerevisiae *[8]. Clearly, however, this information should be considered with caution, since all methods are known to yield a non-negligible amount of noise (false positives) and to miss a fraction of existing interactions (false negatives). The error rate depends strongly on the method, high-throughput and computational methods being less reliable than traditional methods [9].

The network of interactions between proteins is generally represented as an interaction graph, where nodes represent proteins and edges represent pairwise interactions. Graph theory approaches have been applied to describe the topological properties of the network: distribution of node degree (number of incoming and outgoing edges per node), network diameter (average of the shortest distance between pairs of nodes), clustering coefficient (proportion of the potential edges between the neighbors of a node that are effectively observed in the graph). These analyses have led to the observation of some apparently recurrent properties of biological networks: power-law degree distribution, small world, high clustering coefficients, and modularity [10-15].

Beyond these descriptive statistics, an important challenge for modern biology is to understand the relationship between the organization of a network and its function. In particular, it is essential to extract functional modules such as protein complexes [16] or regulatory pathways [17] from global interaction networks.

To achieve this goal, several clustering methods have been applied to the protein interactome graph in order to detect highly connected subgraphs (e.g. [18-34]). These algorithms rely on very different approaches. Each of them requires specifying several parameters, some of which may drastically affect the results. To our knowledge, no systematic study has yet been performed to evaluate and compare these programs. It is thus very difficult for a biologist to estimate the reliability of hypotheses emerging from computer-based analyses of interaction networks.

In this paper we present a systematic quantitative evaluation of the capability of four clustering methods for inferring protein complexes from a network of pairwise protein interactions. The four methods tested here are Markov Clustering (MCL [35,36]), Restricted Neighborhood Search Clustering (RNSC [21]), Molecular Complex Detection (MCODE [19]), and Super Paramagnetic Clustering (SPC [37]). For each program, we sample the parameter space and select optimal parameters. We evaluate the robustness of the programs to false positives and false negatives. The algorithms are then applied to six data sets from high-throughput experiments.

## Results and discussion

### Algorithms

The four algorithms tested here rely on distinct approaches for extracting clusters from the graph (Table 1). We give hereafter a short conceptual description. More information can be found in the supplementary material [see Additional file 1] and original publications.

**Table 1:** Main features of the graph clustering approaches presented in this study.

	**Restricted Neighborhood Search Clustering (RNSC)**	**Markov Clustering (MCL)**	**Molecular Complex Detection (MCODE)**	**Super-paramagnetic clustering (SPC)**
**Type**	Local search cost based	Flow simulation	Local neighbourhood density search	Hierarchical
**Allow multiple assignations**	No	No	Yes	No
**Allow unassigned nodes**	No	No	Yes	No
**Edge-weighted graphs supported**	No	Yes	No	Yes
**First application**	Protein complex prediction	Protein family detection	Protein complex detection	
**Other applications**	/	Identification of ortholog groups, protein complexes, peer-to-peer node clustering, image retrieval, Word Sense Discrimination, molecular pathway discovery, structural domains, ...	/	Image clustering, microarray data clustering, protein complexes detection, protein structure classification, identification of ortholog groups, ...
**Availability**	Upon request			Upon request
**Developper**	King AD	Van Dongen S	Bader GD and Hogue CWV	Blatt M, Wiseman S, Domany E
**References**	[21]	[35]	[19]	[18]

The Markov Cluster algorithm (MCL) [35,36] simulates a flow on the graph by calculating successive powers of the associated adjacency matrix. At each iteration, an *inflation step *is applied to enhance the contrast between regions of strong or weak flow in the graph. The process converges towards a partition of the graph, with a set of high-flow regions (the clusters) separated by boundaries with no flow. The value of the *inflation parameter *strongly influences the number of clusters.

The second algorithm, Restricted Neighborhood Search Clustering (RNSC) [21]), is a cost-based local search algorithm that explores the solution space to minimize a cost function, calculated according to the numbers of intra-cluster and inter-cluster edges. Starting from an initial random solution, RNSC iteratively moves a vertex from one cluster to another if this move reduces the general cost. When a (user-specified) number of moves has been reached without decreasing the cost function, the program ends up.

The third algorithm, Super Paramagnetic Clustering (SPC) [37] is a hierarchical clustering algorithm inspired from an analogy with the physical properties of a ferromagnetic model subject to fluctuation at nonzero temperature. At first, SPC associates a *spin *with each node of the graph. Spins belonging to a highly connected region fluctuate in a correlated fashion and nodes with correlated spins are placed in the same cluster. When the temperature increases, the system becomes less stable and the clusters become smaller.

The fourth method, Molecular Complex Detection (MCODE) [19], detects densely connected regions. First it assigns a weight to each vertex, corresponding to its local neighborhood density. Then, starting from the top-weighted vertex (seed vertex), it recursively moves outward, including in the cluster vertices whose weight is above a given threshold. This threshold corresponds to a user-defined percentage of the weight of the seed vertex.

### Interaction graphs

**Figure 1:**
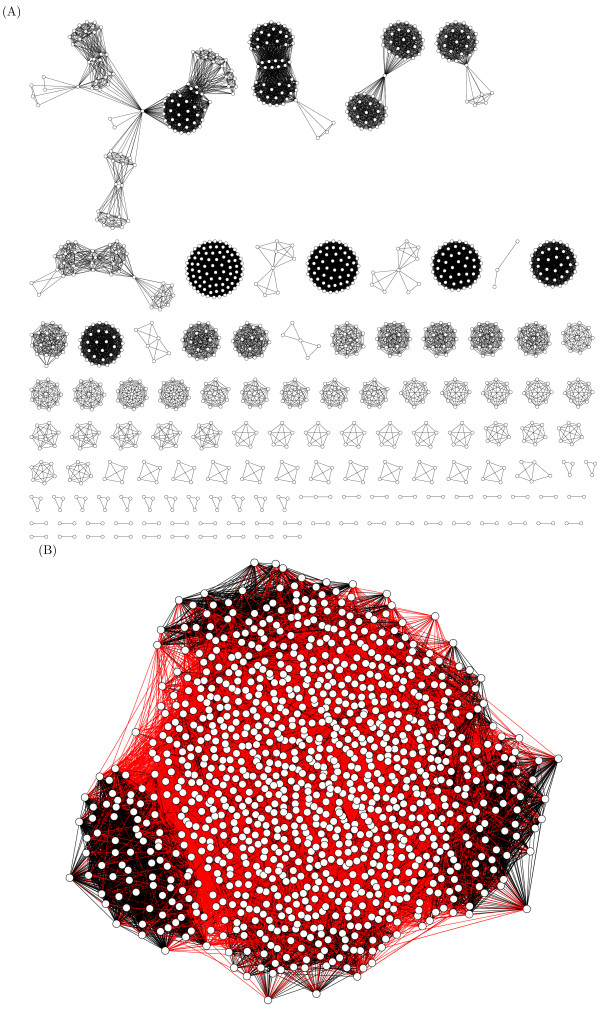
**Graphical representation of interaction networks**. **(A) **Test graph built from the complexes annotated in the MIPS database (high-throughput data were excluded). **(B) **Altered graph *A*_100,40 _with 100% of random edge addition (red) and 40% of random edge removal.

From the collection of protein complexes annotated in the MIPS database [38], we constructed an interaction graph by instantiating a node for each protein, and linking by an edge any two proteins that belong to the same complex. This graph is hereafter referred to as the *test graph*. As depicted in Figure 1A, the structure of the original test graph is almost trivial: most complexes correspond to isolated components. In this test graph each complex is represented as a clique (each protein is connected to each other one). This generally does not reflect the actual complex structure, where each protein is linked to specific partners. Consequently, this original graph is of poor value for evaluating the performances of clustering algorithms on real data sets. This applies particularly to high-throughput data sets, which are generally fragmentary (missing interactions), and noisy (false interactions).

In order to evaluate the robustness of the algorithms to missing and false interactions, we generated 41 *altered graphs *from the original test graph, by combining addition and removal of edges in various proportions. We refer to altered graphs as *A*_*add*,*del*_, where *add *and *del *indicate respectively the percentage of added and deleted edges (percentages with respect to the number of edges in the original test graph).

Figure 1B shows an example of an altered graph *A*_100,40, _with 100% edge addition and 40% edge removal. Another problem of evaluation is that a certain proportion of interacting proteins can be assigned to the same cluster by chance. In order to estimate the random expectation of correct grouping, we built a *random graph *by shuffling the edges between nodes of the test graph. With this type of randomization, each node preserves the same number of links as in the original graph.

We also built 41 *altered random graphs *from the random graph, by randomly adding and removing random edges in the same proportions as for the original test graph.

To each of these 84 graphs (test, altered test, random, altered random), we applied the four algorithms described above, with varying parameter values. As a second way to estimate the random expectation, each clustering result was also randomized so as to obtain a set of *permuted clusters *of the same sizes as those obtained from the test graph or altered graphs.

### Parameter optimization

The quality of a clustering result was evaluated by comparing each cluster with each annotated complex. The *complex-wise sensitivity *(*Sn*) represents the coverage of a complex by its best-matching cluster (the maximal fraction of proteins in the complex found in a common cluster). Reciprocally, the *cluster-wise Positive Predictive Value *(*PPV*) measures how well a given cluster predicts its best-matching complex (see the chapter *Methods *for a detailed description of the matching statistics).

To estimate the overall correspondence between a clustering result (a set of clusters) and the collection of annotated complexes, we computed the weighted means of all *PPV *values (averaged over all clusters) and *Sn *values (averaged over all complexes). The resulting statistics, *clustering-wise PPV *and *clustering-wise Sn*, provide complementary and somewhat contradictory information: when the number of clusters decreases, the *Sn *increases and, in the trivial case where all proteins are grouped in a single cluster, the calculated *Sn *reaches 1. Reciprocally, the *PPV *increases with the number of clusters, reaching 1 in the trivial case where each protein is assigned to one separate cluster. In order to integrate the two statistics, we computed a *geometrical accuracy *(*Acc*), defined as the geometrical mean of the averaged *Sn *and *PPV *values.

Each algorithm has one or more parameters that influence properties such as number of clusters, cluster size, and cluster density (number of intra-cluster edges). For each algorithm we measured the impact of the main parameters on *Sn, PPV *and *Acc *and selected the combination of parameters giving maximal accuracy. This analysis revealed that some parameters have a drastic impact on accuracy, whereas others have a limited effect.

**Figure 2:**
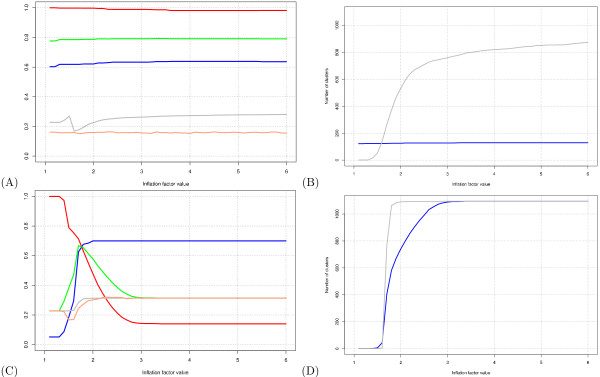
**Impact of the inflation parameter on MCL clustering results**. **(A) **Impact of the inflation parameter on the clustering-wise Sensitivity (*Sn*), Positive Predictive Value (*PPV*) and geometric accuracy (*Acc*). Each curve represents the value of one evaluation statistics (ordinate) as a function of the inflation parameter (abscissa). Color code: *blue *: *Sn*; *red *: *PPV*; *green *: *Acc*; *grey *: geometrical accuracy for the first random control (randomized graph); orange : geometrical accuracy for the second random control (permuted clusters). **(B) **Number of complexes predicted as a function of the inflation factor for the original test graph. Color code: *blue *: test graph; *red *: random graph. **(C) ***Sn*, *PPV *and *Acc *scores obtained with a highly altered graph (*A*_100,40_). **(D) **Number of complexes predicted as a function of the inflation factor for *A*_100,40_.

Let us illustrate in more detail the procedure of parameter selection with the inflation parameter of the MCL algorithm. With the original test graph, interestingly, the effect of this parameter is barely detectable (Figure 2A). Yet this apparent robustness is an artifact due to the trivial structure of the graph. In the MIPS data set used as a reference, most proteins (73%) are members of a single complex, so that most complexes correspond to isolated components in the test graph (Figure 1A) on which the clustering is performed. Consequently, the clustering algorithm tends to define one cluster per connected component, irrespectively of the inflation parameter. Consistently with this interpretation, the number of clusters is almost constant whatever the inflation parameter value (Figure 2B, blue curve). In contrast, when the same algorithm is applied to a randomized graph, the number of clusters increases with the inflation parameter (Figure 2B, gray curve).

The crucial impact of the inflation parameter becomes obvious when MCL is applied to highly altered graphs. For example, for the altered graph *A*_100,40 _(Figure 2C), the increase in inflation causes a decrease in *Sn *(red curve) and an increase in *PPV *(blue curve). These effects are explained by the fact that the number of clusters increases with the inflation parameter (Figure 2D). The optimal tradeoff between *Sn *and *PPV *is obtained for an inflation value of 1.7, and yields an *Acc *of 66% (green curve).

We performed the same analysis and selected the optimal parameter values for each one of the 42 graphs (test and altered), as summarized in Table 2 for the MCL algorithm. Since the optimal parameter values depend on the level of alteration, we cannot view one value as systematically optimal. We chose as a general optimum the most frequent value in this table. This criterion ensures a good robustness to graph alteration (it covers the widest range of graph alterations).

**Table 2:** Optimal values for MCL inflation parameter for the test and altered graphs

% removal\% addition	0	5	10	20	40	80	100
0	3.4	3.1	2.7	2.4	2	1.8	1.8
5	5.7	4	2.6	2	1.9	1.8	1.8
10	2.35	2.2	2.2	2.3	1.8	1.8	1.8
20	1.7	2.2	2.1	2	1.8	1.7	1.8
40	1.8	1.8	1.8	1.9	1.7	1.7	1.7
80	1.3	1.4	1.5	1.5	1.5	1.6	1.6

Note that in the case of the inflation parameter, the most frequent value (1.8) is especially well suited for graphs with a high level of alteration, such as those resulting from high-throughput data. In addition, for the less altered graphs, the accuracy is generally more robust to fluctuations of the inflation (the extreme case of the unaltered test graph shown in Figure 2A,B is discussed above).

**Figure 3:**
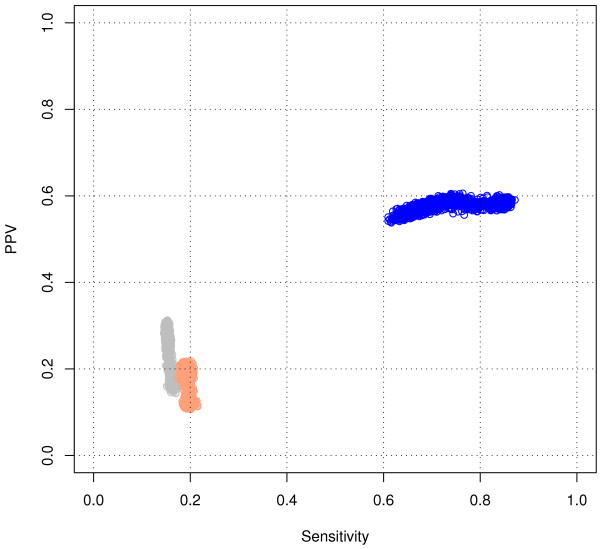
**Impact of the RNSC parameters on the clustering of an altered graph *A*_100,40_**. Each dot represents the clustering-wise *PPV *and *Sn *value for one combination of the seven tested parameters. Color code: *blue *: altered graph *A*_100,20 _(100% random edge addition and 20% of random edge removal); *orange *: randomized graph *R*_100,40_; *grey *: permuted clusters.

For the RNSC algorithm, we tested the impact of 7 parameters on the quality of the clustering. This represents a total of 2,916 combinations of parameter values. Figure 3 displays the *Sn *(abscissa) and *PPV *(ordinate) obtained with the same altered graph as in Figure 1B (*A*_100,40_). Each dot corresponds to one particular combination of parameter values. This figure shows that the RNSC algorithm is remarkably robust to the choice of parameter values: all the results are grouped in a cloud, with an almost constant *PPV *(58%) and a restricted range of *Sn *(between 61% and 87%).

The same analysis was carried out for each parameter of each algorithm. The complete tables of optimal values for the 42 graphs using both Accuracy and Separation (see next section) are available as supplementary material [see Additional file 2 and 3]. Table 3 synthesizes the optimal values obtained for the four tested algorithms. These optimal values were systematically used for the robustness analysis in the next section.

**Table 3:** Optimal parameters

**Algorithm**	**Parameter**	**Optimized for accuracy**	**Optimized for separation**
**MCL**	Inflation	1.8	1.8

**MCODE**	Depth	100	5
	Node score percentage	0	0
	Haircut	TRUE	TRUE
	Fluff	FALSE	FALSE
	Percentage for complex fluffing	0.2	0.9

**RNSC**	Diversification frequency	50	50
	Shuffling diversification length	9	3
	Tabu length	50	50
	Tabu list tolerance	1	1
	Number of experiments	3	3
	Naive stopping tolerance	1	15
	Scaled stopping tolerance	15	15

**SPC**	Number of nearest neighbours	15	10
	Temperature	0.132	0.116

### Robustness analysis

In this analysis, we chose fixed parameter values for each algorithm (Table 3) and analyzed the robustness of the different algorithms to various levels of graph alteration (edge removal and addition).

**Figure 4:**
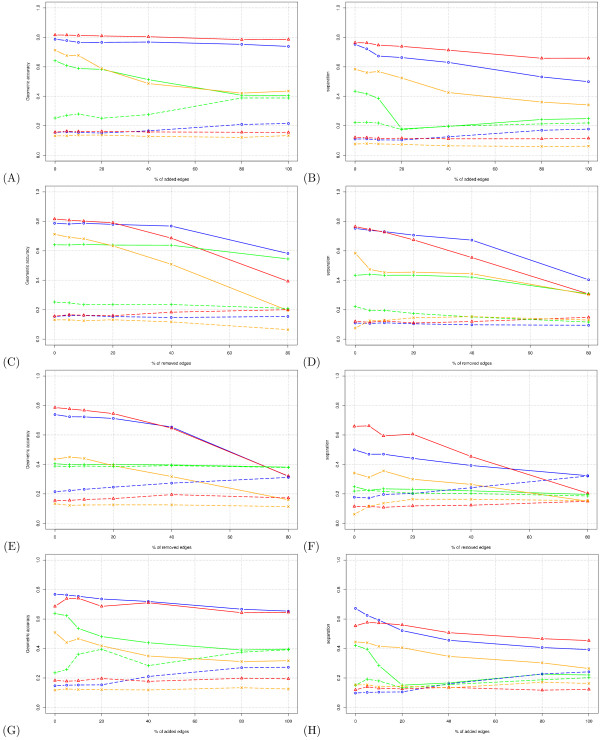
**Robustness of the algorithms to random edge addition and removal**. Each curve represents the value of accuracy (left panels) or separation (right panels). **(A-B) **edge addition to the test graph. **(C-D) **edges removal from the test graph. **(E-F) **Edge removal from an altered graph with 100% of randomly added edges. **(G-H) **Edge addition to an altered test graph with 40% of randomly removed edges. Color code: *blue *: MCL, *red *: RNSC, *orange *: MCODE, *green *: SPC. Dotted lines show the results obtained by permuting the clusters (negative control).

Figure 4A displays the impact of edge addition on the geometric accuracy. Increasing proportions of edges (0%, 5%, 10%, 20%, 40%, 80% and 100%) were randomly added to the test graph. MCL and RNSC are barely affected by addition of up to 100% edges (blue and red curves, respectively). The performances of MCODE and SPC are reasonably good for low values of noise, but drop to 40% when the percentage of added edges increases to 100% (orange and green curves, respectively).

To estimate the random expectation, we performed for each clustering result a permutation test, by shuffling the proteins between clusters. The number of clusters and their respective sizes thus remained unchanged. The geometric accuracy of the permuted clusters is displayed with dotted lines in Figure 4A. For MCL, RNSC and MCODE, the accuracy is relatively stable (between 15% and 22%). For SPC, surprisingly, the accuracy of the permuted clusters progressively increases with the addition of edges, reaching 38% when more than 80% egdes are added. This value almost equals that obtained with the non-random altered graph *A*_100,0_. This illustrates the importance of the permutation test: the test makes it possible to estimate the performance of an algorithm in terms of gains relative to the random expectation. We inspected the clustering result in more detail in order to understand why the program can yield high accuracy values even when clusters are permuted. This effect comes from the fact that, under the chosen conditions, SPC yields a huge cluster of 567 proteins, plus a multitude of very small clusters of 1 or 2 proteins. The effect of the huge cluster is to artificially increase the *Sn*, since a good fraction of each complex is covered by this cluster. Each of the very small clusters yields a high *PPV *: single-element clusters have by definition a *PPV *of 1, and 2-member clusters have a minimal *PPV *of 0.5. This particular distribution of cluster sizes thus creates an artefactual situation by reaching, for two separate reasons, reasonably high scores for both criteria (*Sn *and *PPV*).

In order to circumvent this problem, we defined an additional statistic, which we call *separation*, as the product of the proportion of complex elements found in the cluster by the proportion of cluster elements found in the complex (see Methods for the formula). High separation values indicate a bidirectional correspondence between a cluster and a complex: a maximal value of 1 is reached when a cluster corresponds perfectly with a complex, i.e. when it comprises all of its proteins and nothing more.

The *complex-wise separation *indicates how well a given complex is isolated from the other complexes. The maximal value for complex-wise separation is 1. The simplest way to obtain Sepcoi
 MathType@MTEF@5@5@+=feaafiart1ev1aaatCvAUfKttLearuWrP9MDH5MBPbIqV92AaeXatLxBI9gBaebbnrfifHhDYfgasaacH8akY=wiFfYdH8Gipec8Eeeu0xXdbba9frFj0=OqFfea0dXdd9vqai=hGuQ8kuc9pgc9s8qqaq=dirpe0xb9q8qiLsFr0=vr0=vr0dc8meaabaqaciaacaGaaeqabaqabeGadaaakeaacqWGtbWucqWGLbqzcqWGWbaCdaWgaaWcbaGaem4yamMaem4Ba82aaSbaaWqaaiabdMgaPbqabaaaleqaaaaa@350C@ = 1 is the perfect match, i.e. when all the proteins in the complex are contained in a single cluster, and this cluster does not contain any other protein (Table 4, cluster 1/complex 1). Yet the value of 1 can also be reached if the complex is split into two or more clusters, if each of these clusters contains only members of the complex (Table 4, complex 2 split into clusters 2 and 3). In other words, Sepcoi
 MathType@MTEF@5@5@+=feaafiart1ev1aaatCvAUfKttLearuWrP9MDH5MBPbIqV92AaeXatLxBI9gBaebbnrfifHhDYfgasaacH8akY=wiFfYdH8Gipec8Eeeu0xXdbba9frFj0=OqFfea0dXdd9vqai=hGuQ8kuc9pgc9s8qqaq=dirpe0xb9q8qiLsFr0=vr0=vr0dc8meaabaqaciaacaGaaeqabaqabeGadaaakeaacqWGtbWucqWGLbqzcqWGWbaCdaWgaaWcbaGaem4yamMaem4Ba82aaSbaaWqaaiabdMgaPbqabaaaleqaaaaa@350C@ = 1 indicates that the clustering algorithm separates this complex perfectly from all other complexes (although this complex may be split into several clusters).

**Table 4:** Schematic illustration of a contingency table, and the derived statistics

**Counts**							
*T*	**cluster 1**	**cluster 2**	**cluster 3**	**cluster 4**	**cluster 5**	**sum**	**complex size**

**complex 1**	7	0	0	0	0	7	7
**complex 2**	0	6	8	0	0	14	14
**complex 3**	0	0	0	14	3	17	20
**complex 4**	0	0	0	4	5	9	8

**sum**	7	6	8	18	8	47	
**cluster size**	7	6	8	16	8		

**Positive Predictive Value (PPV)**							

*PPV*	**cluster 1**	**cluster 2**	**cluster 3**	**cluster 4**	**cluster 5**		

**complex 1**	1	0	0	0	0		
**complex 2**	0	1	1	0	0		
**complex 3**	0	0	0	0.78	0.38		
**complex 4**	0	0	0	0.22	0.62		

**cluster-wise PPV**	1	1	1	0.78	0.62		

**Sensitivity**							

*Sn*	**cluster 1**	**cluster 2**	**cluster 3**	**cluster 4**	**cluster 5**	**complex-wise Sn**	

**complex 1**	1	0	0	0	0	1	
**complex 2**	0	0.43	0.57	0	0	0.57	
**complex 3**	0	0	0	0.70	0.15	0.70	
**complex 4**	0	0	0	0.50	0.62	0.62	

**Frequency per row**							

*F*_*row*_	**cluster 1**	**cluster 2**	**cluster 3**	**cluster 4**	**cluster 5**		

**complex 1**	1	0	0	0	0		
**complex 2**	0	0.43	0.57	0	0		
**complex 3**	0	0	0	0.82	0.18		
**complex 4**	0	0	0	0.44	0.56		

**Separation**							

*C*	**cluster 1**	**cluster 2**	**cluster 3**	**cluster 4**	**cluster 5**	**complex-wise separation**	

**complex 1**	1	0	0	0	0	1	
**complex 2**	0	0.43	0.57	0	0	1	
**complex 3**	0	0	0	0.64	0.07	0.71	
**complex 4**	0	0	0	0.10	0.35	0.45	

**cluster-wise separation**	1	0.43	0.57	0.74	0.41		

Clustering-wise sensitivity 0.69

Clustering-wise PPV 0.85

Accuracy 0.77

Average cluster-wise separation 0.63

Average complex-wise separation 0.79

Clustering-wise separation 0.70

Similarly, we defined a *cluster-wise separation*, which indicates how well a given cluster isolates one or several complexes from the other clusters. The maximal value, Sepclj
 MathType@MTEF@5@5@+=feaafiart1ev1aaatCvAUfKttLearuWrP9MDH5MBPbIqV92AaeXatLxBI9gBaebbnrfifHhDYfgasaacH8akY=wiFfYdH8Gipec8Eeeu0xXdbba9frFj0=OqFfea0dXdd9vqai=hGuQ8kuc9pgc9s8qqaq=dirpe0xb9q8qiLsFr0=vr0=vr0dc8meaabaqaciaacaGaaeqabaqabeGadaaakeaacqWGtbWucqWGLbqzcqWGWbaCdaWgaaWcbaGaem4yamMaemiBaW2aaSbaaWqaaiabdQgaQbqabaaaleqaaaaa@3508@ = 1, indicates that a cluster fully and exclusively comprises all the elements of one or several complexes, i.e. it contains all the proteins of the considered complex(es), and no other cluster contains any of these proteins.

The *clustering-wise separation *statistic integrates separation values over all complexes and clusters, and indicates the general correspondence between a clustering result and the set of annotated complexes. Separation is particularly relevant to assessing clustering algorithms like MCODE, which permit assigning a protein to multiple clusters. Under some particular parameter combinations, this program tends to yield highly redundant clusters. Table 5 shows a fragment of the contingency table indicating the number mutual intersections between the 607 clusters obtained from the unaltered test graph. For example, the 50 first rows/columns show a series of imbricated clusters, each resulting from the addition of one node to the preceding cluster. Such strongly overlapping clusters artificially increase the performance, since a set of clusters representing the same complex will be taken into account multiple times in the average *PPV*.

**Table 5:** Mutually overlapping clusters obtained under some parameter conditions with MCODE

cluster\cluster	1	2	3	4	5	...	49	50	51	52	...	102	103	...	607
1	81	80	79	78	77	...	47	46	0	0	...	0	32	...	0
2	80	80	79	78	77	...	47	46	0	0	...	0	32	...	0
3	79	79	79	78	77	...	47	46	0	0	...	0	32	...	0
4	78	78	78	78	77	...	47	46	0	0	...	0	32	...	0
5	77	77	77	77	77	...	47	46	0	0	...	0	32	...	0
...	...	...	...	...	...	...	...	...	...	...	...	...	...	...	...
49	47	47	47	47	47	...	47	46	0	0	...	0	32	...	0
50	46	46	46	46	46	...	46	46	0	0	...	0	32	...	0
51	0	0	0	0	0	...	0	0	46	0	...	0	0	...	0
52	0	0	0	0	0	...	0	0	0	46	...	32	0	...	0
...	...	...	...	...	...	...	...	...	...	...	...	...	...	...	...
102	0	0	0	0	0	...	0	0	0	32	...	32	0	...	0
103	32	32	32	32	32	...	32	32	0	0	...	0	32	...	0
...	...	...	...	...	...	...	...	...	...	...	...	...	...	...	...
607	0	0	0	0	0	...	0	0	0	0	...	0	0	...	3

Cluster-wise separation penalizes this effect by using the marginal sums rather than the cluster size. Thus, if a method generates many redundant clusters, each one intersecting with a given complex, the marginal sum will increase drastically, and *Sep*_*cl *_will be reduced accordingly. Note that the result of Table 5 is not representative of all MCODE conditions: when appropriate parameters are chosen, the level of mutual overlap between clusters is reasonable.

Figure 4B displays the impact of edge addition on clustering-wise separation. The general trends are similar to those revealed by the accuracy curves (Figure 4A), but the random expectation curves are now roughly horizontal for SPC as well as for the other algorithms. We defined a second set of parameters optimized for separation, in the same way as described above for accuracy. These separation-optimized parameters are displayed in Table 3 and were used for all separation curves in this robustness analysis (right panels in Figure 4).

In Figure 4C and 4D, increasing proportions (0%, 5%, 10%, 20%, 40%, and 80%) of edges are randomly removed from the test graph. The general trend is for RNSC and MCL to outperform the other two algorithms under most conditions. RNSC, however, shows a higher sensitivity to edge removal, and its performance strongly decreases when more than 40% of the edges are removed. SPC is quite robust to edge removal, but its performance remains lower than that of MCL under all conditions. Note that this removal experiment is not very indicative of algorithm capability under realistic conditions, because the partitioning of the test graph corresponds almost with complex composition (Figure 1A). Thus, when edges are simply removed, this partitioning is mostly maintained: given the high level of intra-complex connectivity, most complexes remain linked, and no new inter-complex link is created.

In order to obtain a realistic estimate of algorithm robustness, we thus need to combine edge addition and removal. Figure 4E and 4F shows the robustness to edge removal, starting from a graph with 100% edge addition. The performances of all programs are of course reduced as compared to Figures 4C and 4D. In terms of accuracy (Figure 4E), RNSC and MCL show grossly similar behaviours: the accuracy shows a good robustness in the low range of removal percentages (0–40%) but strongly decreases at higher percentages (80%). Yet in terms of separation (Figure 4F), RNSC shows a better performance than MCL at low rates of removal. The separation values of all algorithms drop to their respective levels of the random expectation when 80% of the edges are removed. MCODE and SPC show generally low performance, and are drastically affected by the combination of addition and removal. The performance of SPC is similar to that obtained by selecting random clusters, in terms of both accuracy (Figure 4E) and separation (Figure 4F).

Figures 4G and 4H show the effect of edge addition on graphs from which 40% of the edges had previously been removed. These curves confirm the trends observed in Figures 4A and 4B: MCL and RNSC are weakly affected by edge addition, but as little as 20% edge addition suffices to prevent SPC from identifying the complexes (Figure 4H). MCODE is relatively robust to edge addition, but shows a weaker performance than MCL and RNSC over the whole range of conditions.

### Analysis of data sets obtained in high-throughput experiments

In the previous chapters our evaluations were based on artificial graphs obtained by adding and removing various proportions of edges to a reference network (the MIPS complexes). The next step was to evaluate the capability of these algorithms to extract relevant information from high-throughput data sets. To this end, we downloaded from the GRID database [39] six data sets representing the network of protein interactions in the yeast *Saccharomyces cerevisiae*. Two of these data sets consist of pairs of interacting proteins detected by the two-hybrid technique published respectively by Uetz *et al*. [1] and Ito *et al*. [2]. The four other data sets contain protein complexes characterized by mass spectrometry, published respectively by Gavin *et al*. [4,6], Ho *et al*. [5], and Krogan *et al*. [7] (Table 6). For each of these data sets we built a graph with one node per protein, and one edge per interaction.

**Table 6:** Main features of the four large scale data sets and clustering performances of the algorithms when applied to them

Dataset	Nb nodes	Nb edges	Mean degree	Mean clust coeff		MCL		MCODE		RNSC		SPC	
						real	permuted	real	permuted	real	permuted	real	permuted

Uetz *et al*. [1]	926	865	1.175	0.018	Number of clusters	288		10		48		234	
					Mean nb prot/cluster	3.22		11.2		1.91		3.96	
					Median nb prot/cluster	3		4.5		2		2	
					Largest cluster size	16		53		6		276	
					*Sn*	57.3%	38.6%	84.3%	74.5%	49.4%	36.5%	65.5%	43.3%
					*PPV*	53.8%	45.9%	25.5%	21.6%	59.6%	54.4%	38.0%	38.9%
					*Acc*_*g*_	55.6%	42.3%	54.9%	48.0%	54.5%	45.5%	51.8%	41.1%
					*Sep*_*co*_	23.0%	20.6%	48.9%	62.5%	15.5%	14.8%	19.1%	21.2%
					*Sep*_*cl*_	30.1%	26.9%	2.2%	2.8%	34.3%	32.7%	20.3%	22.6%
					*Sep*	26.3%	23.5%	10.4%	13.3%	23.1%	22.0%	19.7%	21.9%

Ito *et al*. [2]	2937	4038	2.682	0.019	Number of clusters	630		9		1746		410	
					Mean nb prot/cluster	4.66		97.8		1.68		7.16	
					Median nb prot/cluster	3		11		2		2	
					Largest cluster size	157		485		4		1928	
					*Sn*	34.9%	26.0%	66.9%	68.0%	31.4%	24.0%	73.2%	64.6%
					*PPV*	42.7%	38.5%	8.2%	5.8%	63.6%	61.8%	24.3%	23.8%
					*Acc*_*g*_	38.8%	32.2%	37.5%	36.9%	47.5%	42.9%	48.8%	44.2%
					*Sep*_*co*_	12.7%	11.8%	41.6%	33.0%	7.1%	7.0%	11.3%	11.0%
					*Sep*_*cl*_	36.2%	33.9%	1.7%	1.3%	56.7%	55.9%	20.1%	20.4%
					*Sep*	21.4%	20%	8.4%	6.7%	20.1%	19.8%	15.4%	15.0%

Ho *et al*. [5]	1564	3600	4.6	0.029	Number of clusters	314		13		957		63	
					Mean nb prot/cluster	4.98		49.5		1.63		24.8	
					Median nb prot/cluster	3		13		1		3	
					Largest cluster size	34		432		8		1383	
					*Sn*	50.6%	28.2%	81.2%	76.5%	37.0%	27.4%	90.1%	92.1%
					*PPV*	47.1%	35.6%	12.9%	8.5%	61.5%	57.1%	10.4%	8.2%
					*Acc*_*g*_	48.9%	31.9%	47.1%	42.5%	49.3%	42.2%	50.2%	50.2%
					*Sep*_*co*_	22.6%	19%	44.7%	37.2%	11%	10.5%	19.3%	13.8%
					*Sep*_*cl*_	32.3%	27.1%	2.6%	2.2%	48%	45.6%	5.5%	4.0%
					*Sep*	27.0%	22.7%	10.9%	9.0%	23%	21.9%	10.3%	7.4%

Gavin *et al*. [4]	1352	3210	4.7	0.148	Number of clusters	212		27		709		87	
					Mean nb prot/cluster	6.38		32.5		1.91		15.5	
					Median nb prot/cluster	4		7		1		2	
					Largest cluster size	54		414		16		1074	
					*Sn*	74.1%	24.2%	67.0%	51.1%	52.1%	20.8%	91.8%	81.4%
					*PPV*	57.0%	23.9%	20.4%	9.4%	62.0%	46.0%	18.1%	10.7%
					*Acc*_*g*_	65.6%	24.0%	43.7%	30.3%	57.1%	33.4%	54.9%	46.0%
					*Sep*_*co*_	39.4%	17.6%	44.5%	16.1%	14.5%	11.3%	34.4%	15.7%
					*Sep*_*cl*_	38.0%	17.0%	5.5%	2.0%	46.9%	36.5%	13.6%	6.2%
					*Sep*	38.7%	17.3%	15.6%	5.6%	26.1%	20.3%	21.6%	9.8%

Gavin *et al*. [6]	1430	6531	9.1	0.348	Number of clusters	189		39		487		136	
					Mean nb prot/cluster	7.57		40.3		2.94		10.5	
					Median nb prot/cluster	4		9		2		3	
					Largest cluster size	90		697		35		620	
					*Sn*	75.7%	23.7%	58.3%	43.2%	60.8%	20.9%	79.8%	48.4%
					*PPV*	54.3%	21.0%	20.6%	8.0%	63.3%	37.3%	37.0%	16.5%
					*Acc*_*g*_	65.0%	22.4%	39.5%	25.6%	62.1%	29.1%	58.4%	32.4%
					*Sep*_*co*_	38.1%	15.5%	44.7%	15.3%	20.1%	12.9%	34.9%	14.9%
					*Sep*_*cl*_	32.7%	13.3%	7.9%	2.7%	44.5%	28.6%	21.6%	9.2%
					*Sep*	35.3%	14.4%	18.8%	6.4%	29.9%	19.2%	27.4%	11.7%

Krogan *et al*. [7]	2675	7088	5.296	0.146	Number of clusters	813		70		1405		114	
					Mean nb prot/cluster	4.93		28.3		2.1		10.3	
					Median nb prot/cluster	3		5.5		2		3	
					Largest cluster size	50		387		21		1724	
					*Sn*	62.8%	19.8%	56.3%	30.9%	53.1%	19.1%	82.6%	64.0%
					*PPV*	56.2%	33.5%	21.9%	9.7%	63.3%	51.1%	25.4%	17.2%
					*Acc*_*g*_	59.5%	26.7%	39.1%	20.3%	58.2%	35.1%	54.0%	40.6%
					*Sep*_*co*_	20.0%	12.1%	33.2%	13.6%	10.3%	8.7%	20.3%	11.9%
					*Sep*_*cl*_	49.5%	29.9%	8.8%	3.6%	59.6%	50.3%	24.0%	14.1%
					*Sep*	31.5%	19.0%	17.0%	7.0%	24.7%	21.6%	20.9%	12.9%

**Figure 5:**
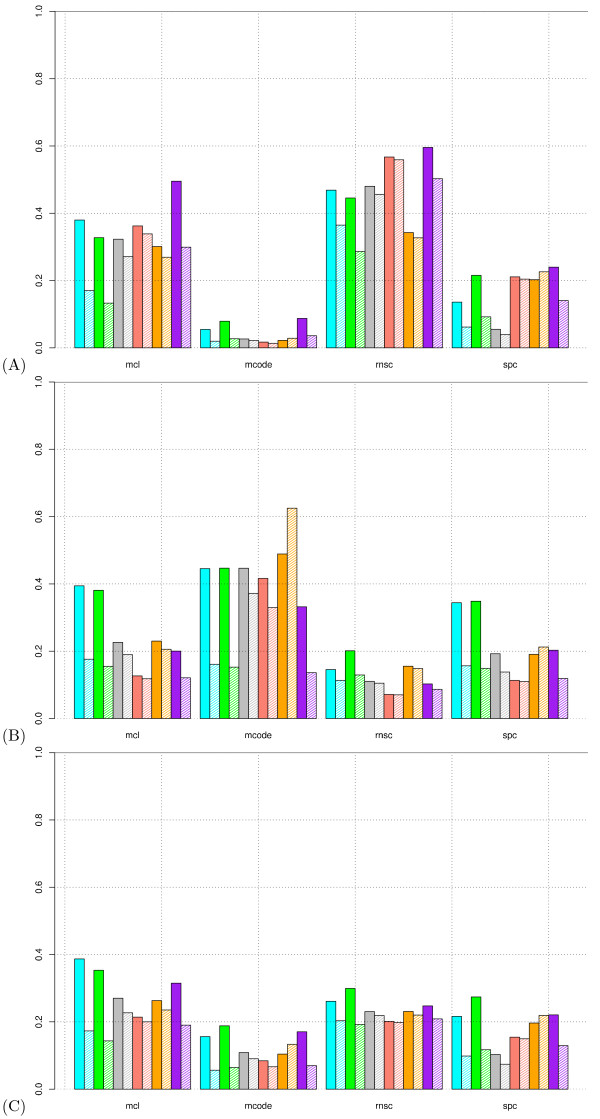
**Application of clustering on high-throughput data sets**. **(A) **Cluster-wise separation. **(B) **Complex-wise separation. **(C) **Clustering-wise separation. **Color code: ***blue *: mass-spectrometry data set from Gavin et al. (2002); *green *: mass-spectrometry data set from Gavin et al. (2006); *grey *: mass-spectrometry data set from Ho et al. (2002); *orange *: two-hybrid data set from Ito et al. (2001); *yellow *: two-hybrid data set from Uetz et al. (2000); *purple *: mas-spectrometry data set from Krogan et al. (2006). Shaded boxes show the results obtained by permuting the clusters (negative control).

We then ran the four clustering algorithms on these graphs, with the optimal parameters determined in the first part of this study. The clusters obtained from these high-throughput networks were compared with the complexes annotated in the MIPS database by computing the same statistics as described above (Table 6, Figure 5). In each case, a negative control was done by calculating the same statistics on permuted clusters (shaded boxes in Figure 5).

Some precautions should be taken before interpreting these results. In particular, it is not trivial to interpret the "positive predictive value", as our reference set is the MIPS collection, filtered to discard any high-throughput result. This collection should by no means be considered exhaustive, since the complexes detected by previous studies represent only a fraction of all existing complexes. High-throughput methods are thus expected to yield many complexes that have not previously been characterized by other methods. Thus, interactions detected by high-throughput methods that are not annotated in MIPS cannot be considered "false positives". The same holds true for cluster-wise separation. Thus, the *PPV *and cluster-wise separation values should be interpreted as an indication of the fraction of high-throughput results which are also detected by other methods and have been annotated in the MIPS so far. In contrast, the sensitivity is likely to yield more directly relevant information, by indicating the fraction of annotated complexes recovered in the clusters obtained from high-throughput data. Bearing in mind these limitations, we may now analyse the data presented in Table 6 and Figure 5.

An important criterion for this analysis is the contrast between the scores reached with the real clustering results and the random expectation estimated with permuted clusters. A look at this contrast already reveals some general characteristics of the data sets. Whatever the clustering method used, the *Sep *values obtained were similar for the real and pemuted clusters in the case of the data sets resulting from two-hybrid experiments [1,2]. This confirms the conclusions of Von Mering *et al*., who compared the positive predictive value (called "accuracy" in their paper) and sensitivity ("coverage") of the interaction graphs obtained by various high-throughput methods [8], and who also observed very poor values for the two-hybrid data sets. The HMS-PCI data set [5] shows a better contrast between real and permuted clusters, but the best results are clearly obtained with the three other mass-spectrometry data sets [4,6,7]. We will thus focus on these three data sets in our comparison of the algorithms.

Compared to the other algorithms, MCODE yields a lower number of clusters, with a higher number of proteins per cluster. It generally yields a moderate sensitivity, a low *PPV*. It is characterized by very weak cluster-wise separation (Figure 5A), contrasting with a very high complex-wise separation (Figure 5B). The resulting clustering-wise separation values (Figure 5C) are lower than for the other algorithms. Despite its relatively weak general performance, MCODE interestingly shows the best performances for the negative control (i.e. the lowest values). This reflects the fact that this algorithm has the capability to discard nodes from the clustering result (unassigned nodes). Apparently, this property enables the program to discard most nodes when a random graph is submitted, but this seems to be at the expense of sensitivity with real interaction graphs.

SPC is characterized by a high sensitivity and a low *PPV*. Yet the high sensitivity is an artifact due to the presence of one mega-cluster, generally accompanied by a multitude of mini-clusters. The asymmetry between cluster sizes is revealed by the differences between the mean and median numbers of proteins per cluster. For Gavin (2002), for example, SPC yields 87 clusters with a mean size of 15.5 but with a median of only 2 proteins per cluster. The mega-cluster includes no less than 1074 proteins and thus comprises most complexes, raising the sensitivity to *Sn = *91.8%. The artificial aspect is indicated by the fact that the permuted clusters also reach a very high score *Sn = *81.4%. As discussed above, this bias is avoided by the separation statistics, which yield relatively low values for SPC. Similar figures are obtained with the other data sets.

For all the data sets, RNSC yields a large number of mini-clusters (the average number of proteins per cluster is typically 2, the median is 1 or 2), plus a few clusters of reasonable size (up to 35 proteins per cluster). It shows a relatively high cluster-wise separation value (Figure 5A) but a lower complex-wise separation value (Figure 5B), resulting in a reasonable tradeoff in terms of clustering-wise separation (Figure 5C). The contrast between real and permuted clusters is low, however, even for the mass-spectrometry data with which other algorithms reach a good contrast.

Finally, MCL clearly outperforms the other algorithms in terms of general performance (Table 6, *Acc, Sep*) and also as regards the contrast between real and permuted clusters (Table 6, Figure 5). This general performance results from a good balance between cluster-wise (Figure 5A) and clustering-wise (Figure 5B) separation.

## Conclusion

We have evaluated the capability of four graph-based clustering algorithms to extract protein complexes from networks of protein-protein interactions. This evaluation has led us to elaborate a testing procedure for the selection of optimal parameters and the analysis of robustness to noise. We have defined new matching statistics called *separation *to circumvent some pitfalls of classical estimators (Sensitivity, PPV, Accuracy). The methodology proposed here could be used as well to assess the capabilities of other clustering algorithms with other data sets.

To study the ability of the tested algorithms to extract protein complexes from an interaction network, we built a test graph from the complexes annotated in the MIPS database.

In a first step we assessed the impact of the parameters of each algorithm, and determined the optimal values for extracting complexes from an interaction network. This analysis shows that under most conditions, RNSC and MCL outperform MCODE and SPC. RNSC is remarkably robust to variations in the choice of parameters, whereas the other algorithms require appropriate tuning in order to yield relevant results. Secondly we assessed the robustness of these programs to noise and to missing information in the data, by randomly adding and removing edges from the test graph. This analysis clearly revealed differences between the algorithms, highlighting the robustness of MCL, and to a lesser extent RNSC, to graph alterations.

We then applied the same four algorithms to interaction networks obtained from six high-throughput studies. This analysis revealed that whatever the algorithm used, some data sets provide insufficient information for extracting the correct protein complexes. An analysis of the more informative data sets confirmed the general superiority of MCL over the three other algorithms tested here.

An important limitation of the present evaluation is that it was performed by naive users. Any algorithm is likely to work better in the hands of its own developer than in those of external users. As we did not participate in the development of any of the tested algorithms, our evaluation may underestimate the capabilities of some of the algorithms tested here. An advantage of such an external evaluation, however, is that the evaluators are not biased by better knowledge of one particular algorithm. Consequently, our evaluation might be biased in favour of algorithms which are more user-friendly, or easier to configure. It thus reflects a compromise between algorithm user-friendliness and efficiency.

Another limitation is that all of our analyses were performed on unweighted graphs, because our reference graph (the MIPS complexes) does not contain any information that would enable us to assign reliability values (weights) to the edges. It should be mentioned that MCL and SPC can deal with weighted graphs and are likely to give better performances if the weights reflect the reliability of the links between proteins [20].

## Methods

### Test graphs

#### Annotated protein complexes

In order to test the ability of each algorithm to extract complexes from a network of binary interactions, we built a graph representing a large collection of experimentally characterized complexes. We collected from the MIPS database the collection of protein complexes annotated for the yeast *Saccharomyces cerevisiae *[40], from which we discarded those resulting from high-throughput experiments [41]. The filtered collection contains some cases of hierarchically related complexes. For example, the complex annotated as "ribosome" includes the small and large ribosomal subunits. In such cases, we discarded the parent complex (ribosome) and retained the sub-complexes only (small and large subunits). The final set comprises 220 complexes. It was converted to a graph where each node represents one polypeptide. A link (edge) was created between each pair of polypeptides involved in a common complex. The resulting graph (referred to as *test graph*) contains 1,095 polypeptides and 12,261 interactions (Figure 1A).

#### Altered graphs

A series of 41 altered graphs was derived from the test graph described above by combining various proportions of random edge deletions (0%, 5%, 10%, 20%, 40%, 80%) and additions (0%, 5%, 10%, 20%, 40%, 80%, 100%). We refer to altered graphs as *A*_*add*,*del *_where *add *and *del *indicate, respectively, the percentage of added and deleted edges.

### Random expectation

The random expectation of clustering results was estimated in two ways: with randomized graphs and permuted clusters.

#### Randomized graphs

A *randomized graph *was obtained by shuffling all the edges of the test graph. This procedure preserves the connectivity of each node while reallocating edges at random. We also generated 41 *altered randomized graphs *by randomly adding edges to and deleting edges from the random graph in the same proportions as for the test graph. We refer to altered randomized graphs as *R*_*add*,*del*, _where *add *and *del *indicate respectively the percentage of added and deleted edges.

#### Permuted clusters

A set of permuted clusters can be obtained from a clustering result by shuffling the associations between proteins and clusters. This randomization procedure preserves cluster sizes. We applied it to each clustering result obtained with the test graph and the altered graphs.

### Matching statistics

Each clustering result was compared with the annotated complexes by building a contingency table, as schematically exemplified in Table 4. Having *n *complexes and *m *clusters, the contingency table *T *is a *n*·*m *matrix where row *i *corresponds to the *i*^*th *^annotated complex, and column *j *to the *j*^*th *^cluster. The value of a cell *T*_*i*,*j *_indicates the number of proteins found in common between complex *i *and cluster *j*. Note that some proteins belong to several complexes, and that with one one algorithm (MCODE), some proteins may be assigned to multiple clusters or not assigned to any cluster. The marginal sums (per row or per column) of the contingency table thus do not always correspond with complex or cluster sizes. For example, cluster 4 in Table 4 contains 16 proteins, but the sum of intersections between this cluster and all complexes is 18, because complexes 3 and 4 have 2 proteins in common. Complex 3 contains 20 proteins, but the sum of its intersections with clusters is 17, because 3 of its proteins are not assigned to any cluster. On the contrary, the fourth complex of Table 4 contains 8 proteins, but there are 9 assignations to clusters in all, because one protein is assigned to two separate clusters.

Sensitivity, positive predictive value (*PPV*), and accuracy are classically used to measure the correspondence between the result of a classification and a reference. We describe hereafter how these concepts can be adapted to measuring the match between a set of protein complexes and a clustering result. As discussed in the text, these statistics can in some particular cases lead to erroneous interpretations. We thus define an additional statistic, which we call *separation*.

#### Sensitivity

Considering the annotated complexes as our reference classification, we define *sensitivity *as the fraction of proteins of complex *i *which are found in cluster *j*.

*Sn*_*i*,*j *_= *T*_*i*,*j*_*/N*_*i*_

In this formula, *N*_*i *_is the number of proteins belonging to complex *i*. We also calculate a *complex-wise sensitivity *Sncoi
 MathType@MTEF@5@5@+=feaafiart1ev1aaatCvAUfKttLearuWrP9MDH5MBPbIqV92AaeXatLxBI9gBaebbnrfifHhDYfgasaacH8akY=wiFfYdH8Gipec8Eeeu0xXdbba9frFj0=OqFfea0dXdd9vqai=hGuQ8kuc9pgc9s8qqaq=dirpe0xb9q8qiLsFr0=vr0=vr0dc8meaabaqaciaacaGaaeqabaqabeGadaaakeaacqWGtbWucqWGUbGBdaWgaaWcbaGaem4yamMaem4Ba82aaSbaaWqaaiabdMgaPbqabaaaleqaaaaa@33B5@ as the maximal fraction of proteins of complex *i *assigned to the same cluster. Sncoi
 MathType@MTEF@5@5@+=feaafiart1ev1aaatCvAUfKttLearuWrP9MDH5MBPbIqV92AaeXatLxBI9gBaebbnrfifHhDYfgasaacH8akY=wiFfYdH8Gipec8Eeeu0xXdbba9frFj0=OqFfea0dXdd9vqai=hGuQ8kuc9pgc9s8qqaq=dirpe0xb9q8qiLsFr0=vr0=vr0dc8meaabaqaciaacaGaaeqabaqabeGadaaakeaacqWGtbWucqWGUbGBdaWgaaWcbaGaem4yamMaem4Ba82aaSbaaWqaaiabdMgaPbqabaaaleqaaaaa@33B5@ reflects the coverage of complex *i *by its best-matching cluster.

Sncoi=maxj=1mSni,j
 MathType@MTEF@5@5@+=feaafiart1ev1aaatCvAUfKttLearuWrP9MDH5MBPbIqV92AaeXatLxBI9gBaebbnrfifHhDYfgasaacH8akY=wiFfYdH8Gipec8Eeeu0xXdbba9frFj0=OqFfea0dXdd9vqai=hGuQ8kuc9pgc9s8qqaq=dirpe0xb9q8qiLsFr0=vr0=vr0dc8meaabaqaciaacaGaaeqabaqabeGadaaakeaacqWGtbWucqWGUbGBdaWgaaWcbaGaem4yamMaem4Ba82aaSbaaWqaaiabdMgaPbqabaaaleqaaOGaeyypa0dcbiGae8xBa0Mae8xyaeMae8hEaG3aa0baaSqaaiabdQgaQjabg2da9iabigdaXaqaaiabd2gaTbaakiabdofatjabd6gaUnaaBaaaleaacqWGPbqAcqGGSaalcqWGQbGAaeqaaaaa@4430@

To characterize the general sensitivity of a clustering result, we compute a *clustering-wise sensitivity *as the weighted average of Sncoi
 MathType@MTEF@5@5@+=feaafiart1ev1aaatCvAUfKttLearuWrP9MDH5MBPbIqV92AaeXatLxBI9gBaebbnrfifHhDYfgasaacH8akY=wiFfYdH8Gipec8Eeeu0xXdbba9frFj0=OqFfea0dXdd9vqai=hGuQ8kuc9pgc9s8qqaq=dirpe0xb9q8qiLsFr0=vr0=vr0dc8meaabaqaciaacaGaaeqabaqabeGadaaakeaacqWGtbWucqWGUbGBdaWgaaWcbaGaem4yamMaem4Ba82aaSbaaWqaaiabdMgaPbqabaaaleqaaaaa@33B5@ over all complexes.

Sn=∑i=1nNiSncoi∑i=1nNi
 MathType@MTEF@5@5@+=feaafiart1ev1aaatCvAUfKttLearuWrP9MDH5MBPbIqV92AaeXatLxBI9gBaebbnrfifHhDYfgasaacH8akY=wiFfYdH8Gipec8Eeeu0xXdbba9frFj0=OqFfea0dXdd9vqai=hGuQ8kuc9pgc9s8qqaq=dirpe0xb9q8qiLsFr0=vr0=vr0dc8meaabaqaciaacaGaaeqabaqabeGadaaakeaacqWGtbWucqWGUbGBcqGH9aqpdaWcaaqaamaaqadabaGaemOta40aaSbaaSqaaiabdMgaPbqabaGccqWGtbWucqWGUbGBdaWgaaWcbaGaem4yamMaem4Ba82aaSbaaWqaaiabdMgaPbqabaaaleqaaaqaaiabdMgaPjabg2da9iabigdaXaqaaiabd6gaUbqdcqGHris5aaGcbaWaaabmaeaacqWGobGtdaWgaaWcbaGaemyAaKgabeaaaeaacqWGPbqAcqGH9aqpcqaIXaqmaeaacqWGUbGBa0GaeyyeIuoaaaaaaa@4A25@

#### Positive predictive value

The positive predictive value is the proportion of members of cluster *j *which belong to complex *i*, relative to the total number of members of this cluster assigned to all complexes.

PPVi,j=Ti,j/∑i=1nTi,j=Ti,j/T.j
 MathType@MTEF@5@5@+=feaafiart1ev1aaatCvAUfKttLearuWrP9MDH5MBPbIqV92AaeXatLxBI9gBaebbnrfifHhDYfgasaacH8akY=wiFfYdH8Gipec8Eeeu0xXdbba9frFj0=OqFfea0dXdd9vqai=hGuQ8kuc9pgc9s8qqaq=dirpe0xb9q8qiLsFr0=vr0=vr0dc8meaabaqaciaacaGaaeqabaqabeGadaaakeaacqWGqbaucqWGqbaucqWGwbGvdaWgaaWcbaGaemyAaKMaeiilaWIaemOAaOgabeaakiabg2da9iabdsfaunaaBaaaleaacqWGPbqAcqGGSaalcqWGQbGAaeqaaOGaei4la8YaaabCaeaacqWGubavdaWgaaWcbaGaemyAaKMaeiilaWIaemOAaOgabeaaaeaacqWGPbqAcqGH9aqpcqaIXaqmaeaacqWGUbGBa0GaeyyeIuoakiabg2da9iabdsfaunaaBaaaleaacqWGPbqAcqGGSaalcqWGQbGAaeqaaOGaei4la8Iaemivaq1aaSbaaSqaaiabc6caUiabdQgaQbqabaaaaa@5161@

*T*_.*j *_is the marginal sum of a colum *j*. As exemplified by the fourth cluster in Table 4, this marginal sum can in some cases differ from the cluster size, because some proteins can belong to several complexes. We also calculate a *cluster-wise positive predictive value *PPVclj
 MathType@MTEF@5@5@+=feaafiart1ev1aaatCvAUfKttLearuWrP9MDH5MBPbIqV92AaeXatLxBI9gBaebbnrfifHhDYfgasaacH8akY=wiFfYdH8Gipec8Eeeu0xXdbba9frFj0=OqFfea0dXdd9vqai=hGuQ8kuc9pgc9s8qqaq=dirpe0xb9q8qiLsFr0=vr0=vr0dc8meaabaqaciaacaGaaeqabaqabeGadaaakeaacqWGqbaucqWGqbaucqWGwbGvdaWgaaWcbaGaem4yamMaemiBaW2aaSbaaWqaaiabdQgaQbqabaaaleqaaaaa@34A4@, which represents the maximal fraction of proteins of cluster *j *found in the same annotated complex. PPVclj
 MathType@MTEF@5@5@+=feaafiart1ev1aaatCvAUfKttLearuWrP9MDH5MBPbIqV92AaeXatLxBI9gBaebbnrfifHhDYfgasaacH8akY=wiFfYdH8Gipec8Eeeu0xXdbba9frFj0=OqFfea0dXdd9vqai=hGuQ8kuc9pgc9s8qqaq=dirpe0xb9q8qiLsFr0=vr0=vr0dc8meaabaqaciaacaGaaeqabaqabeGadaaakeaacqWGqbaucqWGqbaucqWGwbGvdaWgaaWcbaGaem4yamMaemiBaW2aaSbaaWqaaiabdQgaQbqabaaaleqaaaaa@34A4@ reflects the reliability with which cluster *j *predicts that a protein belongs to its best-matching complex.

PPVclj=maxi=1nPPVi,j
 MathType@MTEF@5@5@+=feaafiart1ev1aaatCvAUfKttLearuWrP9MDH5MBPbIqV92AaeXatLxBI9gBaebbnrfifHhDYfgasaacH8akY=wiFfYdH8Gipec8Eeeu0xXdbba9frFj0=OqFfea0dXdd9vqai=hGuQ8kuc9pgc9s8qqaq=dirpe0xb9q8qiLsFr0=vr0=vr0dc8meaabaqaciaacaGaaeqabaqabeGadaaakeaacqWGqbaucqWGqbaucqWGwbGvdaWgaaWcbaGaem4yamMaemiBaW2aaSbaaWqaaiabdQgaQbqabaaaleqaaOGaeyypa0dcbiGae8xBa0Mae8xyaeMae8hEaG3aa0baaSqaaiabdMgaPjabg2da9iabigdaXaqaaiabd6gaUbaakiabdcfaqjabdcfaqjabdAfawnaaBaaaleaacqWGPbqAcqGGSaalcqWGQbGAaeqaaaaa@4612@

To characterize the general PPV of a clustering result as a whole, we compute a *clustering-wise PPV *as the weighted average of PPVclj
 MathType@MTEF@5@5@+=feaafiart1ev1aaatCvAUfKttLearuWrP9MDH5MBPbIqV92AaeXatLxBI9gBaebbnrfifHhDYfgasaacH8akY=wiFfYdH8Gipec8Eeeu0xXdbba9frFj0=OqFfea0dXdd9vqai=hGuQ8kuc9pgc9s8qqaq=dirpe0xb9q8qiLsFr0=vr0=vr0dc8meaabaqaciaacaGaaeqabaqabeGadaaakeaacqWGqbaucqWGqbaucqWGwbGvdaWgaaWcbaGaem4yamMaemiBaW2aaSbaaWqaaiabdQgaQbqabaaaleqaaaaa@34A4@ over all clusters.

PPV=∑j=1mT.jPPVclj∑j=1mT.j
 MathType@MTEF@5@5@+=feaafiart1ev1aaatCvAUfKttLearuWrP9MDH5MBPbIqV92AaeXatLxBI9gBaebbnrfifHhDYfgasaacH8akY=wiFfYdH8Gipec8Eeeu0xXdbba9frFj0=OqFfea0dXdd9vqai=hGuQ8kuc9pgc9s8qqaq=dirpe0xb9q8qiLsFr0=vr0=vr0dc8meaabaqaciaacaGaaeqabaqabeGadaaakeaacqWGqbaucqWGqbaucqWGwbGvcqGH9aqpdaWcaaqaamaaqadabaGaemivaq1aaSbaaSqaaiabc6caUiabdQgaQbqabaGccqWGqbaucqWGqbaucqWGwbGvdaWgaaWcbaGaem4yamMaemiBaW2aaSbaaWqaaiabdQgaQbqabaaaleqaaaqaaiabdQgaQjabg2da9iabigdaXaqaaiabd2gaTbqdcqGHris5aaGcbaWaaabmaeaacqWGubavdaWgaaWcbaGaeiOla4IaemOAaOgabeaaaeaacqWGQbGAcqGH9aqpcqaIXaqmaeaacqWGTbqBa0GaeyyeIuoaaaaaaa@4DEB@

#### Accuracy

The *geometric accuracy *(*Acc*) indicates the tradeoff between sensitivity and predictive value. It is obtained by computing the geometrical geometrical mean of the *Sn *and the *PPV*.

Acc=Sn⋅PPV
 MathType@MTEF@5@5@+=feaafiart1ev1aaatCvAUfKttLearuWrP9MDH5MBPbIqV92AaeXatLxBI9gBaebbnrfifHhDYfgasaacH8akY=wiFfYdH8Gipec8Eeeu0xXdbba9frFj0=OqFfea0dXdd9vqai=hGuQ8kuc9pgc9s8qqaq=dirpe0xb9q8qiLsFr0=vr0=vr0dc8meaabaqaciaacaGaaeqabaqabeGadaaakeaacqWGbbqqcqWGJbWycqWGJbWycqGH9aqpdaGcaaqaaiabdofatjabd6gaUjabgwSixlabdcfaqjabdcfaqjabdAfawbWcbeaaaaa@39DB@

The advantage of taking the geometric rather than arithmetic mean is that it yields a low score when either the *Sn *or the *PPV *metric is low. High accuracy values thus require a high performance for both criteria. It is of particular importance to use the geometric accuracy, as the arithmetic mean would give a false idea of quality in the trivial cases where all proteins are assigned to a single cluster (*Sn *= 1 ⇒ *Acc*_*arithm *_> 0.5) or where, on the contrary, each protein is assigned to a single-element cluster (*PPV *= 1 ⇒ *Acc*_*arithm *_> 0.5).

#### Separation

The contingency table indicates the absolute frequency of intersections between complexes and clusters. From these values, we derive relative frequencies with respect to the marginal sums, either per row (Frowi,j
 MathType@MTEF@5@5@+=feaafiart1ev1aaatCvAUfKttLearuWrP9MDH5MBPbIqV92AaeXatLxBI9gBaebbnrfifHhDYfgasaacH8akY=wiFfYdH8Gipec8Eeeu0xXdbba9frFj0=OqFfea0dXdd9vqai=hGuQ8kuc9pgc9s8qqaq=dirpe0xb9q8qiLsFr0=vr0=vr0dc8meaabaqaciaacaGaaeqabaqabeGadaaakeaacqWGgbGrdaWgaaWcbaGaemOCaiNaem4Ba8Maem4DaC3aaSbaaWqaaiabdMgaPjabcYcaSiabdQgaQbqabaaaleqaaaaa@3608@) or per column (Fcoli,j
 MathType@MTEF@5@5@+=feaafiart1ev1aaatCvAUfKttLearuWrP9MDH5MBPbIqV92AaeXatLxBI9gBaebbnrfifHhDYfgasaacH8akY=wiFfYdH8Gipec8Eeeu0xXdbba9frFj0=OqFfea0dXdd9vqai=hGuQ8kuc9pgc9s8qqaq=dirpe0xb9q8qiLsFr0=vr0=vr0dc8meaabaqaciaacaGaaeqabaqabeGadaaakeaacqWGgbGrdaWgaaWcbaGaem4yamMaem4Ba8MaemiBaW2aaSbaaWqaaiabdMgaPjabcYcaSiabdQgaQbqabaaaleqaaaaa@35D4@).

Frowi,j=Ti,j∑j=1mTi,j
 MathType@MTEF@5@5@+=feaafiart1ev1aaatCvAUfKttLearuWrP9MDH5MBPbIqV92AaeXatLxBI9gBaebbnrfifHhDYfgasaacH8akY=wiFfYdH8Gipec8Eeeu0xXdbba9frFj0=OqFfea0dXdd9vqai=hGuQ8kuc9pgc9s8qqaq=dirpe0xb9q8qiLsFr0=vr0=vr0dc8meaabaqaciaacaGaaeqabaqabeGadaaakeaacqWGgbGrdaWgaaWcbaGaemOCaiNaem4Ba8Maem4DaC3aaSbaaWqaaiabdMgaPjabcYcaSiabdQgaQbqabaaaleqaaOGaeyypa0ZaaSaaaeaacqWGubavdaWgaaWcbaGaemyAaKMaeiilaWIaemOAaOgabeaaaOqaamaaqadabaGaemivaq1aaSbaaSqaaiabdMgaPjabcYcaSiabdQgaQbqabaaabaGaemOAaOMaeyypa0JaeGymaedabaGaemyBa0ganiabggHiLdaaaaaa@47C9@

Fcoli,j=Ti,j∑i=1nTi,j=PPVi,j
 MathType@MTEF@5@5@+=feaafiart1ev1aaatCvAUfKttLearuWrP9MDH5MBPbIqV92AaeXatLxBI9gBaebbnrfifHhDYfgasaacH8akY=wiFfYdH8Gipec8Eeeu0xXdbba9frFj0=OqFfea0dXdd9vqai=hGuQ8kuc9pgc9s8qqaq=dirpe0xb9q8qiLsFr0=vr0=vr0dc8meaabaqaciaacaGaaeqabaqabeGadaaakeaacqWGgbGrdaWgaaWcbaGaem4yamMaem4Ba8MaemiBaW2aaSbaaWqaaiabdMgaPjabcYcaSiabdQgaQbqabaaaleqaaOGaeyypa0ZaaSaaaeaacqWGubavdaWgaaWcbaGaemyAaKMaeiilaWIaemOAaOgabeaaaOqaamaaqadabaGaemivaq1aaSbaaSqaaiabdMgaPjabcYcaSiabdQgaQbqabaaabaGaemyAaKMaeyypa0JaeGymaedabaGaemOBa4ganiabggHiLdaaaOGaeyypa0JaemiuaaLaemiuaaLaemOvay1aaSbaaSqaaiabdMgaPjabcYcaSiabdQgaQbqabaaaaa@4FF0@

Note that the frequency per column is identical to the PPV defined above. The frequency per row, on the contrary, can differ from the sensitivity for some algorithms, if the algorithm permits assigning a protein to multiple clusters (Table 4, complex 4), or leaving some proteins unassigned (Table 4, complex 3). In such cases, the frequency per row provides a more drastic criterion than the sensitivity defined above.

We define the *separation *as the product of column-wise and row-wise frequencies.

Sepi,j=Fcoli,j⋅Frowi,j
 MathType@MTEF@5@5@+=feaafiart1ev1aaatCvAUfKttLearuWrP9MDH5MBPbIqV92AaeXatLxBI9gBaebbnrfifHhDYfgasaacH8akY=wiFfYdH8Gipec8Eeeu0xXdbba9frFj0=OqFfea0dXdd9vqai=hGuQ8kuc9pgc9s8qqaq=dirpe0xb9q8qiLsFr0=vr0=vr0dc8meaabaqaciaacaGaaeqabaqabeGadaaakeaacqWGtbWucqWGLbqzcqWGWbaCdaWgaaWcbaGaemyAaKMaeiilaWIaemOAaOgabeaakiabg2da9iabdAeagnaaBaaaleaacqWGJbWycqWGVbWBcqWGSbaBdaWgaaadbaGaemyAaKMaeiilaWIaemOAaOgabeaaaSqabaGccqGHflY1cqWGgbGrdaWgaaWcbaGaemOCaiNaem4Ba8Maem4DaC3aaSbaaWqaaiabdMgaPjabcYcaSiabdQgaQbqabaaaleqaaaaa@4A43@

The separation is comprised between 0 and 1. The maximal value *Sep*_*i*,*j *_= 1 indicates a perfect and exclusive correspondence between complex *j *and cluster *i*: it indicates that the cluster contains all the members of the complex and only them (Table 4, complex 1 and cluster 1). In addition, the separation statistic deals efficiently with multiple assignations. It penalizes cases where proteins of a given complex are assigned to multiple clusters, by using row sums rather than complex sizes.

The *complex-wise separation *Sepcoi
 MathType@MTEF@5@5@+=feaafiart1ev1aaatCvAUfKttLearuWrP9MDH5MBPbIqV92AaeXatLxBI9gBaebbnrfifHhDYfgasaacH8akY=wiFfYdH8Gipec8Eeeu0xXdbba9frFj0=OqFfea0dXdd9vqai=hGuQ8kuc9pgc9s8qqaq=dirpe0xb9q8qiLsFr0=vr0=vr0dc8meaabaqaciaacaGaaeqabaqabeGadaaakeaacqWGtbWucqWGLbqzcqWGWbaCdaWgaaWcbaGaem4yamMaem4Ba82aaSbaaWqaaiabdMgaPbqabaaaleqaaaaa@350C@ is calculated as the sum of separation values for a given complex *i*.

Sepcoi=∑j=1mSepi,j
 MathType@MTEF@5@5@+=feaafiart1ev1aaatCvAUfKttLearuWrP9MDH5MBPbIqV92AaeXatLxBI9gBaebbnrfifHhDYfgasaacH8akY=wiFfYdH8Gipec8Eeeu0xXdbba9frFj0=OqFfea0dXdd9vqai=hGuQ8kuc9pgc9s8qqaq=dirpe0xb9q8qiLsFr0=vr0=vr0dc8meaabaqaciaacaGaaeqabaqabeGadaaakeaacqWGtbWucqWGLbqzcqWGWbaCdaWgaaWcbaGaem4yamMaem4Ba82aaSbaaWqaaiabdMgaPbqabaaaleqaaOGaeyypa0ZaaabCaeaacqWGtbWucqWGLbqzcqWGWbaCdaWgaaWcbaGaemyAaKMaeiilaWIaemOAaOgabeaaaeaacqWGQbGAcqGH9aqpcqaIXaqmaeaacqWGTbqBa0GaeyyeIuoaaaa@44B8@

Reciprocally, we calculate a *cluster-wise separation*, which reflects the concentration of one or several complexes within a given cluster.

Sepclj=∑i=1nSepi,j
 MathType@MTEF@5@5@+=feaafiart1ev1aaatCvAUfKttLearuWrP9MDH5MBPbIqV92AaeXatLxBI9gBamXvP5wqSXMqHnxAJn0BKvguHDwzZbqegyvzYrwyUfgarqqtubsr4rNCHbGeaGqiA8vkIkVAFgIELiFeLkFeLk=iY=Hhbbf9v8qqaqFr0xc9pk0xbba9q8WqFfeaY=biLkVcLq=JHqVepeea0=as0db9vqpepesP0xe9Fve9Fve9GapdbaqaaeGacaGaaiaabeqaamqadiabaaGcbaGaem4uamLaemyzauMaemiCaa3aaSbaaSqaaiabdogaJjabdYgaSnaaBaaameaacqWGQbGAaeqaaaWcbeaakiabg2da9maaqahabaGaem4uamLaemyzauMaemiCaa3aaSbaaSqaaiabdMgaPjabcYcaSiabdQgaQbqabaaabaGaemyAaKMaeyypa0JaeGymaedabaGaemOBa4ganiabggHiLdaaaa@54F3@

To estimate a clustering result as a whole, clustering-wise *Sep*_*co *_and *Sep*_*cl *_values are computed as the averages of Sepcoi
 MathType@MTEF@5@5@+=feaafiart1ev1aaatCvAUfKttLearuWrP9MDH5MBPbIqV92AaeXatLxBI9gBaebbnrfifHhDYfgasaacH8akY=wiFfYdH8Gipec8Eeeu0xXdbba9frFj0=OqFfea0dXdd9vqai=hGuQ8kuc9pgc9s8qqaq=dirpe0xb9q8qiLsFr0=vr0=vr0dc8meaabaqaciaacaGaaeqabaqabeGadaaakeaacqWGtbWucqWGLbqzcqWGWbaCdaWgaaWcbaGaem4yamMaem4Ba82aaSbaaWqaaiabdMgaPbqabaaaleqaaaaa@350C@ over all complexes, and of Sepclj
 MathType@MTEF@5@5@+=feaafiart1ev1aaatCvAUfKttLearuWrP9MDH5MBPbIqV92AaeXatLxBI9gBaebbnrfifHhDYfgasaacH8akY=wiFfYdH8Gipec8Eeeu0xXdbba9frFj0=OqFfea0dXdd9vqai=hGuQ8kuc9pgc9s8qqaq=dirpe0xb9q8qiLsFr0=vr0=vr0dc8meaabaqaciaacaGaaeqabaqabeGadaaakeaacqWGtbWucqWGLbqzcqWGWbaCdaWgaaWcbaGaem4yamMaemiBaW2aaSbaaWqaaiabdQgaQbqabaaaleqaaaaa@3508@ over all clusters, respectively.

Sepco=∑i=1nSepcoin
 MathType@MTEF@5@5@+=feaafiart1ev1aaatCvAUfKttLearuWrP9MDH5MBPbIqV92AaeXatLxBI9gBaebbnrfifHhDYfgasaacH8akY=wiFfYdH8Gipec8Eeeu0xXdbba9frFj0=OqFfea0dXdd9vqai=hGuQ8kuc9pgc9s8qqaq=dirpe0xb9q8qiLsFr0=vr0=vr0dc8meaabaqaciaacaGaaeqabaqabeGadaaakeaacqWGtbWucqWGLbqzcqWGWbaCdaWgaaWcbaGaem4yamMaem4Ba8gabeaakiabg2da9maalaaabaWaaabmaeaacqWGtbWucqWGLbqzcqWGWbaCdaWgaaWcbaGaem4yamMaem4Ba82aaSbaaWqaaiabdMgaPbqabaaaleqaaaqaaiabdMgaPjabg2da9iabigdaXaqaaiabd6gaUbqdcqGHris5aaGcbaGaemOBa4gaaaaa@4515@

Sepcl=∑j=1mSepcljm
 MathType@MTEF@5@5@+=feaafiart1ev1aaatCvAUfKttLearuWrP9MDH5MBPbIqV92AaeXatLxBI9gBaebbnrfifHhDYfgasaacH8akY=wiFfYdH8Gipec8Eeeu0xXdbba9frFj0=OqFfea0dXdd9vqai=hGuQ8kuc9pgc9s8qqaq=dirpe0xb9q8qiLsFr0=vr0=vr0dc8meaabaqaciaacaGaaeqabaqabeGadaaakeaacqWGtbWucqWGLbqzcqWGWbaCdaWgaaWcbaGaem4yamMaemiBaWgabeaakiabg2da9maalaaabaWaaabmaeaacqWGtbWucqWGLbqzcqWGWbaCdaWgaaWcbaGaem4yamMaemiBaW2aaSbaaWqaaiabdQgaQbqabaaaleqaaaqaaiabdQgaQjabg2da9iabigdaXaqaaiabd2gaTbqdcqGHris5aaGcbaGaemyBa0gaaaaa@4509@

We then compute the *geometrical separation *(*Sep*) as the geometrical mean of *Sep*_*co *_and *Sep*_*cl*_.

Sep=Sepco⋅Sepcl
 MathType@MTEF@5@5@+=feaafiart1ev1aaatCvAUfKttLearuWrP9MDH5MBPbIqV92AaeXatLxBI9gBaebbnrfifHhDYfgasaacH8akY=wiFfYdH8Gipec8Eeeu0xXdbba9frFj0=OqFfea0dXdd9vqai=hGuQ8kuc9pgc9s8qqaq=dirpe0xb9q8qiLsFr0=vr0=vr0dc8meaabaqaciaacaGaaeqabaqabeGadaaakeaacqWGtbWucqWGLbqzcqWGWbaCcqGH9aqpdaGcaaqaaiabdofatjabdwgaLjabdchaWnaaBaaaleaacqWGJbWycqWGVbWBaeqaaOGaeyyXICTaem4uamLaemyzauMaemiCaa3aaSbaaSqaaiabdogaJjabdYgaSbqabaaabeaaaaa@4195@

### Computation

Clustering was performed on a PC cluster of 40 nodes. Statistical treatments were done and figures made with the freeware statistical package *R *[42]. Graphical representations of the interaction networks were done with CytoScape, an open-source, platform-independent environment for the visualization and analysis of biological networks [43,44].

Graphs of protein interactions were manipulated using the Java classes developed by the aMAZE group [45,46].

## Authors' contributions

SB collected the data, built the graphs, tested the algorithms, and ran the evaluation procedures. JvH conceived of the study and participated in its design, coordination, and in defining the matching statistics. Both authors were equally involved in writing the manuscript.

## Supplementary Material

Additional File 1 Supplementary information about the algorithms Click here for file

Additional File 2 Optimal accuracy parameter values Click here for file

Additional File 3 Optimal separation parameter values. These files and supplementary figures are also available on . Click here for file
