# Supplementary material for: Evaluation of clustering algorithms for protein-protein interaction networks
Source: BMC Bioinformatics. 2006 Nov 6;7:488. doi: 10.1186/1471-2105-7-488 (PMC1637120; doi:10.1186/1471-2105-7-488)
Supplement: Additional File 1 — Supplementary information about the algorithms [file 1471-2105-7-488-S1.pdf]

Evaluation of clustering algorithms for protein-protein interaction networks - supplementary material

Sylvain Brohï¿½ Jacques van Helden

July 25, 2006

Contents

1 Description of the algorithms used in this study 1
1.1 MCL . . . 1
1.2 RNSC . . . 2
1.3 Super paramagnetic clustering . . . 2
1.4 MCODE . . . 3
1.4.1 Vertex weighting . . . 3
1.4.2 Molecular complex prediction . . . 3
1.4.3 Post-processing . . . 4

1 Description of the algorithms used in this study

1.1 MCL

The Markov Cluster algorithm (MCL) is based on random walks on a graph, uses simple algebraic operations on its associated matrix, and does not require a priori knowledge about an underlying cluster structure. As the matrix operations are very simple, the computational time of this program is very low. The MCL algorithm uses the notion of random walk for the retrieval of cluster structure in a graph. In a random walk, at each node the direction to be followed is given by chance.

Imagine a vast collection of random walks, all starting from the same point. Walkers will in general follow different paths. An observer floating high above them will see a flow: the crowd slowly swirls and disperses. Once inside a dense region (with many edges inside), a random walker has little chance to get out. The idea behind MCL is very simple. Simulate many random walks (or flow) within the whole graph, and strengthen flow where it is already strong, and weaken it where it is weak. By repeating the process, an underlying cluster structure will gradually become visible. The process ends up with a number of regions with strong internal flow (clusters), separated by dry boundaries with no flow.

Mathematically, flow is simulated by algebraic operations on the stochastic (Markov) matrix associated with the graph. Flow can be expanded by computing powers of this matrix. Let's G be a graph. The associated Markov matrix associated to it is defined by normalizing all columns of the adjacency matrix of G. Note that each node presents a self-loop (for sake of efficiency). This Markov matrix then represents how each node is attracted to the others. Values contained in this matrix are called transition values. At the first step, we can state that each node is equally attracted to all of its neighbours or at each node, one moves to each of its neighbours

with equal probability. For any Markov matrix N, the powers $N(i)$ have a limit (which may be cyclic). When this limit is reached, no node seems attracted mainly by another one.

However, the initial iterands seems to exhibit a behaviour where transition value are relatively high if the two concerned nodes are located in the same dense region. To increase this effect, one operation, called the *inflation*, is added to the process. At each step, the algorithm will thus change transition values such that preferred neighbours are further favoured and less popular neighbour are demoted. The way to achieve this is by raising all the entries of a certain column to a certain power (the inflation factor) and rescaling the columns to have sum 1 again. The inflation factor must be greater than 1, and the greater this factor, the greater the number of clusters. The matrix obtained after squaring, scaling and inflating a large number of time tends to an equilibrium state with delivers the cluster structure of the graph indicating clearly which nodes are clearly linked to each others.

1.2 RNSC

RNSC is a cost-based local search algorithm that works to minimize a cost function in the solution space. The basic principle underlying local search is to start from an initial candidate solution and then, iteratively, to make moves from one candidate solution to another of the candidate solutions from its direct neighbourhood. The moves are based on local information, and continue until a termination condition is met. Starting from an initial random or user input clustering, RNSC searches a better clustering using a simple integer-valued cost function, the naive cost function. This naive cost function calculates the sum for each vertex i

- the number of neighbours of i that are not in the same cluster
- the number of vertices that are in the same cluster than i but not connected to it.;

This sum is divided by 2. For an optimal clustering, these values have to be low for all vertices. When a user-defined number of moves has been done without decreasing the naive cost function, the program shifts to the real-value scaled cost function which also takes into account the size of the cluster during the calculations.

At each iteration, one node is moved from one cluster to another. If this move decreased the naive or the scaled cost function, this move (considered as an *intensification move*) is conserved.

In order to avoid local minima, a user-defined number of nodes are moved to random clusters according to a user-defined frequency.

In order to avoid cycling back to the previously explored partitioning, a list of vertices that may not move is maintained. The size of this list and the number of time a vertex must be in the list before being considered as *non movable* are user-defined.

1.3 Super paramagnetic clustering

This hierarchical clustering algorithm has been used in numerous domains and is based on an analogy to the physics of inhomogeneous ferromagnets. It uses this analogy to find tightly connected clusters on large graphs. Consider a collection of sites $\{a, b, ..., n\}$. In a ferromagnet, each pair of site (i, j) presents a non-zero interaction energy between them. We can thus form an interaction graph $([n], E)$ where the sites are the nodes and the weighted edges, the interaction energy on each pair. Following different possible algorithms, the Potts Model attributes a spin value $(s_1, s_2, ..., s_n)$ to each node in order to decrease the following energy cost function.

$$H[\{s\}] = -\sum_{\langle i,j \rangle} J_{ij} d_{s_i, s_j}$$

In this function, J is some monotonically decreasing function with the distance $\|x_i - x_j\|$, so that the closer two points to each other, the more they like to belong to the same class. d is 1 when s_i and s_j are equal, 0 otherwise. In order to give value to all sites, some different algorithm coexist. One of these is the Swendsen-Wang algorithm (Swendsen and Wang, 1987), which is as follows. In a particular state of a spin system there are clusters of spins. A spin cluster consists of a set of spins, each of which is a nearest neighbour to at least one other spin in the cluster, with all spins having the same spin value. The Swendsen-Wang process consists of two parts:

(1) Creation of "virtual" spin clusters :

If two spins are connected by an open lattice bond and those spins have the same value then a "virtual" bond is created between them with probability $1 - e^{-J/T}$, where T is the temperature. Thereby each spin cluster is partitioned into (smaller) virtual spin clusters.

(2) The second part of the Swendsen-Wang process :

For each virtual spin cluster, select a spin value at random (from among all possible spin values) and assign that spin value to all spins in the virtual cluster. This process is then repeated.

Domany et co-workers used an analogy to this Potts model in order to cluster graphs. In this approach, every node i on the graph is assigned a label s_i, which takes an integer spin value which corresponds to a Potts spin variable. Two nodes i and j are neighbours if j is one of the k nearest points to i (k, being specified by user) and they interact with strength J_{ij} inversely proportional to the distance between them. This distance is the weight value on each edge. At non-zero temperature, the system is subject to equilibration making spins fluctuate (Swendsen-Wang process). By detecting spin-spin correlation at each temperature, vertices are placed in the same cluster. By varying the temperature from 0 to 1, the clustering resolution changes and a hierarchical clustering is obtained. At temperature 0, all vertices are placed in the same cluster which splits as temperature increases.

1.4 MCODE

MCODE uses a vertex-weighting scheme based on the clustering coefficient which measures the density of the neighbourhood of a vertex. The density of an unweighted graph is the proportion of possible edge that are present. The density of a graph $G = (V, E)$ with number of vertices $\|V\|$ and number of edges $\|E\|$ is defined as $\|E\|$ divided by the theoretical maximum number of edges possible for the graph $\|E\|_{max}$, ($\|E\|_{max}$ being equal to $\frac{\|V\|(\|V\|+1)}{2}$, if the graph contains loops and $\frac{\|V\|(\|V\|-1)}{2}$ if the graph contains no loop). The MCODE algorithm operates in three steps :

1.4.1 Vertex weighting

The highest k-core of a graph is the central most densely connected subgraph. The first stage of MCODE consists in weighting all vertices using the local network density using the highest k-core of vertex neighbourhood.

1.4.2 Molecular complex prediction

Starting from the highest valued vertex, MCODE will try to compute clusters. It will include in the cluster the vertices whose weight is above a given threshold, which is a given percentage from the weight of the seed vertex. This percentage is the VWP parameter (vertex weight percentage). A vertex is not checked more than once, because at this stage, clusters cannot overlap. This process stops once no more vertices can be added to the complex based on the given threshold and is repeated for the next highest valued vertex.

1.4.3 Post-processing

Complexes are first filtered to contain graph with minimum degree 2. If the algorithm is run with the fluff option, then the neighbours of the clusters are added to them if their value is higher than a given fluff parameter (between 0 and 1). If the haircut option is run, the cluster is 2-cored, meaning that the vertices linked to cluster by a single edge are removed. The resulting clusters are then scored and ranked. The score is defined as the product of the cluster subgraph density and the number of vertices.
