# Supplementary material for: Evaluation of clustering algorithms for protein-protein interaction networks
Source: BMC Bioinformatics. 2006 Nov 6;7:488. doi: 10.1186/1471-2105-7-488 (PMC1637120; doi:10.1186/1471-2105-7-488)
Supplement: Additional File 2 — Optimal accuracy parameter values [file 1471-2105-7-488-S2.pdf]

Evaluation of clustering algorithms for protein-protein interaction networks - Optimal parameter values

Sylvain Brohée and Jacques van Helden

July 27, 2006

All the values in these table correspond to the mode of the parameter values giving the best accuracy results for a given alteration degree of the MIPS graph.

1 MCL

1.1 Optimal inflation values

rm/ad	0	5	10	20	40	80	100
0	3.4	3.1	2.7	2.4	2	1.8	1.8
5	5.7	4	2.6	2	1.9	1.8	1.8
10	2.35	2.2	2.2	2.3	1.8	1.8	1.8
20	1.7	2.2	2.1	2	1.8	1.7	1.8
40	1.8	1.8	1.8	1.9	1.7	1.7	1.7
80	1.3	1.4	1.5	1.5	1.5	1.6	1.6

2 MCODE

2.1 Depth from source node to limit complex

rm/ad	0	5	10	20	40	80	100
0	5	5	5	5	5	5	5
5	12.5	5	5	5	5	5	5
10	60	5	5	5	5	5	5
20	41.66	5	5	5	60	5	5
40	5	5	20	5	5	5	5
80	5	60	5	20	1	1	1

2.2 Neighbour density percentage threshold for complex fluffing

rm/ad	0	5	10	20	40	80	100
0	0.75	0.49	0.49	0.558	0.55	0.5583	0.585
5	0.75	0.49	0.9	0.49	0.75	0.75	0.5
10	0.15	0.9	0.9	0.49	0.58	0.5	0.5
20	0.75	0.9	0.75	0.75	0.5	0.5	0.585
40	0.15	0.9	0.5	0.5	0.2	0.2	0.2
80	0.1	0.2	0.1	0.2	0.58	0.2	0.53

2.3 Fluff complexes

rm/ad	0	5	10	20	40	80	100
0	TRUE	FALSE	FALSE	FALSE	FALSE	FALSE	FALSE
5	TRUE	FALSE	TRUE	FALSE	TRUE	TRUE	TRUE
10	TRUE	TRUE	TRUE	FALSE	FALSE	TRUE	TRUE
20	TRUE	TRUE	TRUE	TRUE	TRUE	TRUE	FALSE
40	TRUE	TRUE	TRUE	TRUE	TRUE	TRUE	TRUE
80	TRUE	TRUE	TRUE	TRUE	FALSE	TRUE	FALSE

2.4 Give complex a haircut

rm/ad	0	5	10	20	40	80	100
0	TRUE/FALSE	FALSE	FALSE	FALSE	TRUE	FALSE	TRUE
5	FALSE	TRUE/FALSE	FALSE	FALSE	TRUE	TRUE	TRUE
10	FALSE	TRUE	FALSE	FALSE	TRUE	TRUE	TRUE
20	FALSE	FALSE	TRUE	TRUE	TRUE	TRUE	TRUE
40	FALSE	TRUE	TRUE	TRUE	TRUE	TRUE	FALSE
80	FALSE	TRUE	TRUE	TRUE	TRUE	TRUE	TRUE

2.5 Node score percentage threshold for core complex expansion

rm/ad	0	5	10	20	40	80	100
0	0.5	0.05	0	0	0.02	0	0
5	0.1	0.2	0.1	0.1	0.01	0.05	0.01
10	0.2	0.2	0.2	0.2	0.2	0	0.01
20	0.2	0.2	0.2	0.2	0.05	0.01	0.2
40	0.5	0.5	0.2	0.05	0	0	0
80	0.5	0.1	0	0.02	0.5	0.2	0.2

3 RNSC

3.1 Shuffling diversification length

rm/ad	0	5	10	20	40	80	100
0	8	3	9	9	9	5	9
5	3	5.7142	5	9	9	5	9
10	5	6.73333	5	5	5	5	9
20	5.44	4.86666	3	9	9	5	3
40	3	3	3	5	9	5	9
80	9	9	5	9	5	3	5

3.2 Diversification frequency

rm/ad	0	5	10	20	40	80	100
0	20	20	10	10	10	10	10
5	10	47.8571428571429	10	10	10	20	20
10	17.5	22	20	10	10	10	10
20	48.889	45.33	50	50	50	50	50
40	50	50	20	50	50	20	20
80	20	20	50	20	50	10	50

3.3 Number of experiments

rm/ad	0	5	10	20	40	80	100
0	10	3	1	3	3	10	3
5	3	2.9283	1	10	3	3	10
10	6.5	6.8	10	10	3	3	10
20	3.740	6	1	1	1	3	3
40	10	1	3	3	10	10	10
80	10	1	3	3	3	1	1

3.4 Naive stopping tolerance

rm/ad	0	5	10	20	40	80	100
0	20	20	10	10	10	10	10
5	10	47.8571428571429	10	10	10	20	20
10	17.5	22	20	10	10	10	10
20	48.888	45.33	50	50	50	50	50
40	50	50	20	50	50	20	20
80	20	20	50	20	50	10	50

3.5 Scaled stopping tolerance

rm/ad	0	5	10	20	40	80	100
0	12.5	15	5	5	1	5	15
5	15	5.714	15	15	15	1	1
10	12.5	11.666	15	15	15	15	1
20	15	11.666	5	15	5	15	15
40	15	15	15	15	15	5	15
80	15	15	15	15	5	15	15

3.6 Tabu length

rm/ad	0	5	10	20	40	80	100
0	52.5	50	25.5	1	1	50	10
5	50	40.7142857142857	1	1	1	100	10
10	7.75	29.13	10	10	100	10	50
20	72.962	61.333	50	100	10	50	50
40	100	100	10	50	10	1	1
80	100	10	50	100	50	1	10

3.7 Tabu list tolerance

rm/ad	0	5	10	20	40	80	100
0	1	3	1	1	1	3	5
5	3	1	3	1	3	5	5
10	4.5	3.266	1	3	1	1	3
20	1	1.4	1	1	1	1	1
40	1	3	1	1	1	5	5
80	5	3	1	5	5	1	1

4 SPC

4.1 K nearest neighbour parameter

rm/ad	0	5	10	20	40	80	100
0	15	20	15	20	20	85	20
5	85	15	15	20	15	85	20
10	65	20	15	10	15	65	20
20	85	15	10	15	15	55	15
40	150	10	8	10	30	55	15
80	150	7	7	105	65	10	15

4.2 Temperature parameter value

rm/ad	0	5	10	20	40	80	100
0	0.024	0.084	0.116	0.136	0.132	0.124	0.172
5	0.024	0.084	0.116	0.128	0.132	0.132	0.164
10	0.028	0.096	0.116	0.124	0.128	0.124	0.172
20	0.028	0.092	0.12	0.136	0.132	0.128	0.188
40	0.02	0.108	0.124	0.128	0.132	0.12	0.172
80	0.032	0.132	0.124	0.128	0.116	0.152	0.144
