# Supplementary material for: Evaluation of clustering algorithms for protein-protein interaction networks
Source: BMC Bioinformatics. 2006 Nov 6;7:488. doi: 10.1186/1471-2105-7-488 (PMC1637120; doi:10.1186/1471-2105-7-488)
Supplement: Additional File 3 — Optimal separation parameter values. These files and supplementary figures are also available on . [file 1471-2105-7-488-S3.pdf]

Evaluation of clustering algorithms for protein-protein interaction networks - Optimal parameter values

Sylvain Brohée and Jacques van Helden

August 17, 2006

All the values in these table correspond to the mode of the parameter values giving the best separation results for a given alteration degree of the MIPS graph.

1 MCL

1.1 Optimal inflation values

	0	5	10	20	40	80	100
0	5.75	2.7	2.4	2.1	1.9	1.8	1.8
5	2.5	2.5	2.2	2	1.9	1.8	1.8
10	2.35	2.2	2.2	2	1.8	1.7	1.8
20	1.7	2	2.1	1.9	1.8	1.7	1.8
40	1.8	1.8	1.8	1.7	1.7	1.7	1.8
80	1.3	1.5	6	6	5.4	4.4	1.8

2 MCODE

2.1 Depth from source node to limit complex

	0	5	10	20	40	80	100
0	100	5	5	5	5	5	5
5	100	5	5	5	5	5	5
10	60	5	5	5	5	5	5
20	60	5	5	5	5	5	5
40	60	60	5	60	5	5	5
80	20	5	60	5	1	1	1

2.2 Neighbour density percentage threshold for complex fluffing

	0	5	10	20	40	80	100
0	0.49	0.49	0.49	0.49	0.558	0.558	0.5857
5	0.49	0.49	0.49	0.49	0.558	0.5583	0.58
10	0.49	0.49	0.49	0.49	0.558	0.558	0.585
20	0.49	0.49	0.55	0.5	0.585	0.586	0.5857
40	0.49	0.55	0.5583	0.5857	0.586	0.575	0.575
80	0.49	0.5	0.585	0.5857	0.575	0.2	0.53

2.3 Fluff complexes

	0	5	10	20	40	80	100
0	FALSE	FALSE	FALSE	FALSE	FALSE	FALSE	FALSE
5	FALSE	FALSE	FALSE	FALSE	FALSE	FALSE	FALSE
10	FALSE	FALSE	FALSE	FALSE	FALSE	FALSE	FALSE
20	FALSE	FALSE	FALSE	FALSE	FALSE	FALSE	FALSE
40	FALSE	FALSE	FALSE	FALSE	FALSE	FALSE	FALSE
80	FALSE	1	FALSE	FALSE	FALSE	TRUE	FALSE

2.4 Give complex a haircut

	0	5	10	20	40	80	100
0	NA	TRUE	TRUE	TRUE	TRUE	TRUE	TRUE
5	TRUE/FALSE	TRUE	TRUE	TRUE	TRUE	TRUE	TRUE
10	FALSE	TRUE	TRUE	TRUE	TRUE	TRUE	TRUE
20	TRUE/FALSE	TRUE	TRUE	TRUE	TRUE	TRUE	TRUE
40	FALSE	TRUE	TRUE	TRUE	TRUE	TRUE	TRUE
80	TRUE	TRUE	TRUE	TRUE	TRUE	TRUE	TRUE

2.5 Node score percentage threshold for core complex expansion

	0	5	10	20	40	80	100
0	0.5	0.01	0	0	0.02	0	0
5	0.5	0.2	0.1	0.1	0.05	0	0
10	0.5	0.2	0.2	0	0	0	0
20	0.5	0.2	0	0	0	0	0
40	0.5	0	0	0	0	0	0
80	0.95	0.005	0.01	0.01	0	0	0

3 RNSC

3.1 Shuffling diversification length

	0	5	10	20	40	80	100
0	9	9	9	9	9	9	9
5	9	9	9	9	9	9	9
10	9	9	9	9	9	9	9
20	9	9	9	9	9	9	9
40	9	9	9	9	9	9	9
80	9	9	9	9	9	9	9

3.2 Diversification frequency

	0	5	10	20	40	80	100
0	10	10	10	10	10	10	10
5	10	10	10	10	10	10	10
10	10	10	10	10	10	10	10
20	10	10	10	10	10	10	10
40	10	10	10	10	10	10	10
80	10	10	10	10	10	10	10

3.3 Number of experiments

	0	5	10	20	40	80	100
0	10	10	3	10	1	1	1
5	10	3	1	10	1	3	1
10	3	1	1	1	3	3	1
20	3	1	3	10	10	1	1
40	3	1	1	3	3	10	10
80	3	3	1	1	1	3	10

3.4 Naive stopping tolerance

	0	5	10	20	40	80	100
0	10	10	10	10	10	10	10
5	10	10	10	10	10	10	10
10	10	10	10	10	10	10	10
20	10	10	10	10	10	10	10
40	10	10	10	10	10	10	10
80	10	10	10	10	10	10	10

3.5 Scaled stopping tolerance

	0	5	10	20	40	80	100
0	15	1	5	15	5	1	1
5	5	15	5	15	5	15	5
10	5	15	15	5	5	15	5
20	5	15	1	5	5	15	1
40	5	5	1	5	5	1	5
80	1	5	1	15	1	5	15

3.6 Tabu length

	0	5	10	20	40	80	100
0	10	50	1	100	50	10	50
5	100	1	1	1	50	10	100
10	1	100	1	10	50	10	50
20	50	1	10	1	1	50	100
40	1	100	1	1	10	10	100
80	1	10	50	100	1	100	100

3.7 Tabu list tolerance

	0	5	10	20	40	80	100
0	1	1	1	1	3	5	5
5	3	3	3	5	1	3	1
10	5	5	3	5	3	1	5
20	3	5	1	3	1	5	1
40	3	5	3	3	1	3	3
80	3	5	1	3	1	5	1

4 SPC

4.1 K nearest neighbour parameter

	0	5	10	20	40	80	100
0	15	15	10	10	30	130	65
5	105	15	10	10	10	35	150
10	85	10	8	10	15	85	55
20	85	15	8	10	30	30	105
40	55	10	8	10	15	85	85
80	150	150	20	20	150	150	30

4.2 Temperature parameter value

	0	5	10	20	40	80	100
0	0.024	0.084	0.12	0.132	0.14	0.156	0.12
5	0.016	0.08	0.116	0.128	0.148	0.124	0.12
10	0.016	0.104	0.116	0.128	0.132	0.152	0.116
20	0.02	0.092	0.116	0.136	0.144	0.116	0.116
40	0.016	0.108	0.124	0.128	0.132	0.168	0.112
80	0.032	0.26	0.236	0.184	0.132	0.108	0.112
